# Host-directed novel mechanistic insights of doxorubicin reveal its efficacy against drug-resistant HSV-1 underscoring risks with oncolytic virotherapy

**DOI:** 10.1016/j.drup.2026.101362

**Published:** 2026-01-29

**Authors:** Pankaj Sharma, Divya Kapoor, Sudhanshu Kumar Singh, Xiang Shen, Chandrashekhar D. Patil, Deepak Shukla

**Affiliations:** a Department of Ophthalmology and Visual Sciences, University of Illinois Chicago, Chicago, IL, USA; b Department of Microbiology and Immunology, University of Illinois Chicago, Chicago, IL, USA; c Department of Bioengineering, University of Illinois at Chicago, Chicago, IL 60607, USA

**Keywords:** Herpes simplex virus type 1 (HSV-1), Doxorubicin, PI3K-AKT-mTOR pathway, Antiviral therapy, Drug repurposing, Nucleoside analog synergy

## Abstract

**Background:**

Herpes simplex virus type 1 (HSV-1) infects approximately four billion people worldwide, and the emergence of drug-resistant strains has reduced the effectiveness of existing antivirals. Targeting host pathways exploited by HSV-1 represents an attractive strategy for developing resistance-refractory antivirals.

**Methods:**

We evaluated the antiviral potential of doxorubicin, an FDA-approved anticancer drug, against HSV-1 using *in vitro* cell culture systems, an *ex vivo* porcine corneal model, and an *in vivo* murine ocular infection model. Viral replication, host signaling pathways, and combinatorial interactions with nucleoside analogs were systematically assessed.

**Results:**

Doxorubicin potently inhibited HSV-1 replication at sub-cytotoxic concentrations by suppressing the host PI3K-AKT-mTOR signaling axis, a pathway required for viral entry and productive replication. Antiviral activity was observed against laboratory-adapted strains as well as clinical acyclovir-resistant HSV-1 isolates. Pharmacological modulation of PI3K-AKT signaling, pathway activation kinetics, and studies in doxorubicin-resistant cells confirmed a host-directed mechanism. Doxorubicin exhibited strong synergy with nucleoside analog antivirals, enabling dose reduction without loss of efficacy. While inhibition of PI3K-AKT signaling constrained productive replication of both wild-type and oncolytic HSV-1, these effects were context-dependent and relevant to therapeutic settings that rely on robust viral replication.

**Conclusions:**

This study identifies PI3K-AKT pathway inhibition as a novel host-directed antiviral mechanism underlying doxorubicin’s activity against HSV-1, demonstrates its synergistic potential with nucleoside analogs, and provides mechanistic insight into raising concerns over oncolytic HSV-based therapies. Collectively, these findings highlight the potential of localized, host-targeted strategies for managing drug-resistant HSV-1 infections.

## 1. Introduction

Herpes simplex virus type 1 (HSV-1) is a widespread human pathogen that causes lifelong infections and significant morbidity, yet it also serves as the backbone for several oncolytic virotherapy platforms due to its cytolytic capacity and genetic tractability. Symptoms of herpetic infection include orolabial lesions, genital ulcers, herpetic keratitis, encephalitis, and disseminated neonatal disease (Fredrikson et al., 2024). HSV-1 remains the leading cause of infectious blindness worldwide, with herpetic keratitis resulting in significant morbidity due to corneal scarring and vision loss (Su et al., 2024). Despite its global burden, therapeutic options for HSV-1 infections remain limited. Most currently approved antivirals, including acyclovir (ACV) and trifluridine (TFT), target viral DNA polymerase activity, offering effective short-term control but failing to prevent viral latency, reactivation, or the emergence of drug-resistant strains (Rybak-Wolf et al., 2023; Kapoor et al., 2024; Topalis et al., 2018).

Prolonged antiviral therapy, especially in immunocompromised individuals, has led to the increasing detection of ACV-resistant HSV-1 strains, posing serious clinical challenges. Treatment options for resistant infections remain restricted, typically involving alternative nucleoside analogs or second-line agents such as foscarnet, which are associated with substantial toxicity (Bacon et al., 2003; Schalkwijk et al., 2022). Furthermore, long-term and progressively enhanced use of nucleoside analogs is linked to cumulative adverse effects, including nephrotoxicity and ocular complications, underscoring the urgent need for new antiviral strategies that disrupt viral replication through alternative, host-directed mechanisms, offering not only the potential for reduced resistance but also the opportunity to develop synergistic antiviral combinations, which, unlike in the case of HIV, are currently lacking for HSV-1 (Whitley et al., 1986; Eron et al., 1995; Stahl and Mailles, 2019).

HSV-1 exploits host cellular pathways at multiple stages of its life cycle including viral entry, genome replication, and virion release, to establish productive infection. Among these, the phosphoinositide 3-kinase (PI3K)-AKT-mTOR signaling axis plays a central role by promoting viral internalization, inhibiting apoptosis, and supporting viral protein synthesis (Buchkovich et al., 2008; Suryawanshi et al., 2021). HSV-1 also underpins a growing class of oncolytic herpesvirus therapies (oHSVs) genetically engineered strains designed to selectively replicate in tumor cells, induce cytolysis, and stimulate anti-tumor immune responses. While these therapies show promise, particularly in combination with chemotherapeutic agents like doxorubicin, the nature of their interaction remains a critical yet underexplored area. Although many studies highlight synergistic effects, where chemotherapy enhances virus-mediated tumor killing, the possibility that chemotherapy may impair oHSV replication has received comparatively little attention.

Here, we demonstrate that doxorubicin, an FDA-approved chemotherapeutic anti-cancer agent, (Johnson-Arbor and Dubey, 2025; Liu et al., 2025; Gabizon et al., 2016) exhibits potent, broad-spectrum antiviral activity against HSV-1. We show that doxorubicin disrupts HSV-1 infection by inhibiting the PI3K-AKT-mTOR signaling axis. Our data establishes doxorubicin as a potent antiviral, offering a safer, more effective chemotherapeutic option for patients at risk of herpesvirus complications, particularly with reduced doses of standard antivirals to minimize toxicity. Results also suggest extra caution when combining doxorubicin with oncolytic herpesvirus therapy, as potential interactions may reduce cancer-targeting efficacy.

## 2. Results

### 2.1. Doxorubicin exhibits potent anti-HSV-1 activity

**Fig. 1.**
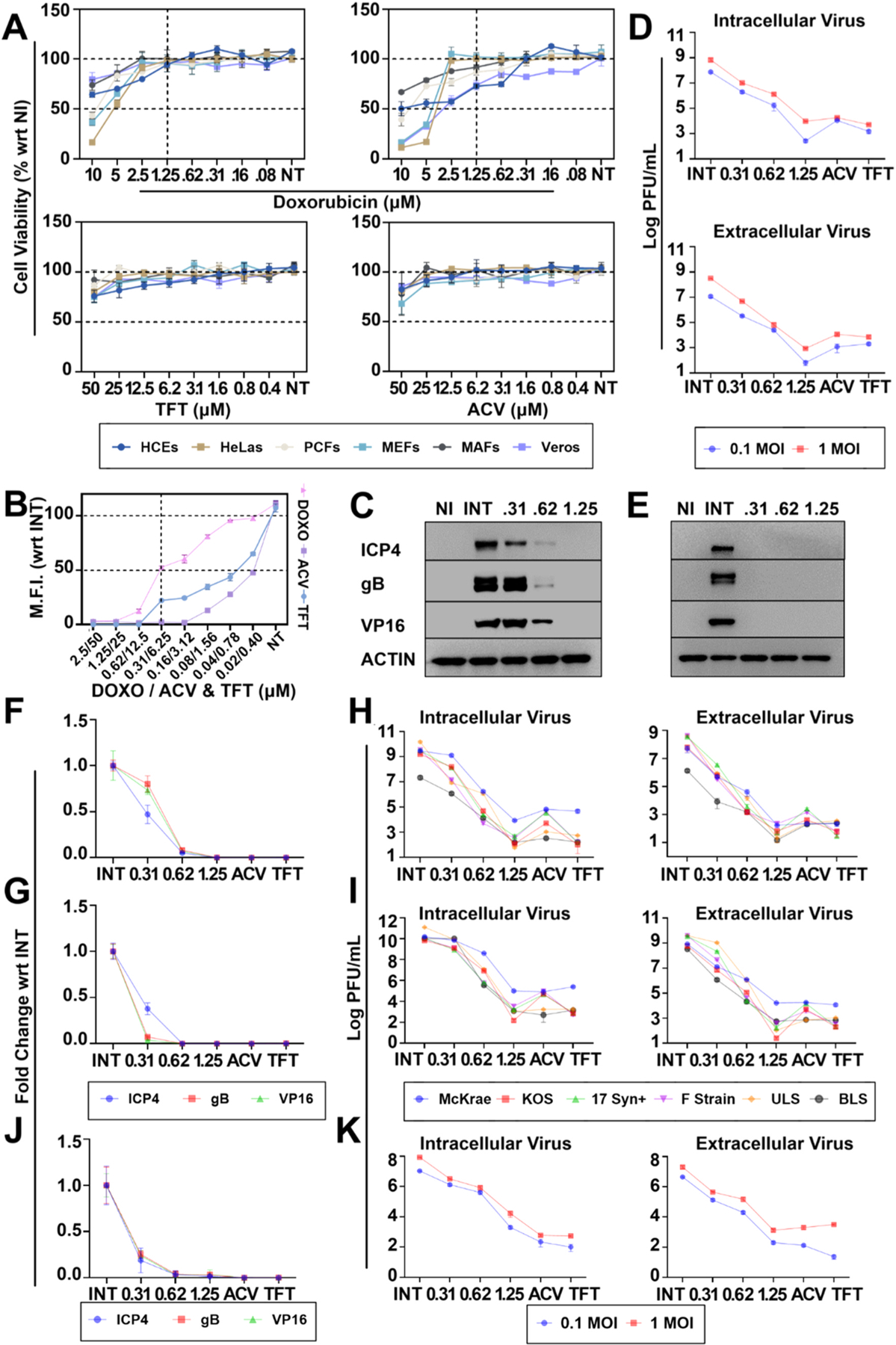
Therapeutic application of doxorubicin suppresses HSV-1 infection under cell culture conditions. (A) Selectivity index (SI) analysis depicting the cytotoxicity of doxorubicin at 24 (left) and 48 h (right) post-treatment, and of acyclovir (ACV) and trifluridine (TFT) at 24 h, across a panel of primary and immortalized cell lines. Immortalized cell lines included human corneal epithelial cells (HCEs), HeLa cells, mouse embryonic fibroblasts (MEFs), and Vero cells, whereas primary cell types comprised pig corneal fibroblast (PCF) cells and mouse adult fibroblast (MAF) cells. (B) Comparative IC_50_ values of doxorubicin, ACV, and TFT in HCE cells infected with HSV-1 K26-GFP at an MOI of 0.1, based on quantification of fluorescence intensity. (C) Immunoblot analysis showing dose-dependent suppression of viral proteins ICP4, VP16, and gB in HCE cells following doxorubicin treatment. (D) Dose-dependent reduction in both intracellular and extracellular viral titers in HeLa cells infected with HSV-1 KOS-WT at low (0.1 MOI) and high (1.0 MOI) multiplicities of infection following doxorubicin treatment. Statistical analysis using one-way ANOVA revealed **** (p < 0.0001) significance for all treatment groups compared to the infected non-treated (INT) control. (E) Immunoblot analysis showing dose-dependent suppression of HSV-1 proteins ICP4, VP16, and gB in HeLa cells following doxorubicin treatment. (F, G) Quantitative PCR (qPCR) analysis demonstrating significant inhibition of viral transcripts (ICP4, VP16, gB) in HCE (F) and HeLa (G) cells upon doxorubicin treatment. All treatments showed **** significance (p < 0.0001) by one-way ANOVA compared to the INT group. (H) Plaque assays showing significant reduction in viral titers across HSV-1 laboratory strains (McKrae, 17 Syn^+^, KOS-WT, F strain) and clinical isolates (ULS, BLS) following doxorubicin treatment in HCE cells at low infection (0.1 MOI). All treatments showed **** significance (p < 0.0001) by one-way ANOVA compared to the INT group. (I) Plaque assays showing reduced titers of both intracellular and extracellular virus across the same HSV-1 strains and clinical isolates following doxorubicin treatment in HCE cells at high infection (1.0 MOI). Statistical analysis revealed **** significance (p < 0.0001) for all treated groups compared to the INT group. (J) qPCR analysis confirming suppressed expression of oncolytic HSV-1 transcripts (ICP4, VP16, gB) in HCE cells after doxorubicin treatment. All treatments were statistically significant (****, p < 0.0001) versus the INT group by one-way ANOVA. (K) Plaque assay quantification demonstrating marked reduction in oncolytic HSV-1 titers in HCE cells at both low (0.1 MOI) and high (1.0 MOI) infection conditions following doxorubicin treatment. All treatments showed **** significance (p < 0.0001) compared to the INT group by one-way ANOVA. All data, except for immunoblot analyses (C and E), represent the mean ± SEM of three independent biological replicates (n = 3).

Our initial rationale for investigating doxorubicin in the context of HSV-1 infection stemmed from its well-characterized role as a DNA intercalating agent, given that HSV-1 is a double-stranded DNA virus. We hypothesized that doxorubicin may interfere with viral replication by targeting the viral genome or associated host pathways. As a preliminary step, we evaluated the cytotoxicity of doxorubicin in human corneal epithelial (HCE) cells across a range of concentrations and identified 2.5 μM as a well-tolerated dose for subsequent experiments (Fig. 1A). We infected HCE cells with a GFP-expressing HSV-1 strain (K26 GFP) and treated them with varying concentrations of doxorubicin, using ACV and TFT as standard antiviral controls. GFP fluorescence intensity revealed that doxorubicin exhibited a significantly lower IC_50_ compared to both ACV and TFT (Fig. 1B), indicating unexpectedly potent antiviral activity (Fig. 1C).

To confirm that this antiviral effect was independent of doxorubicin’s known anticancer activity, we extended the analysis to HeLa cells, a cervical epithelial cancer cell line. Following validation of doxorubicin tolerability, HeLa cells displayed a comparable antiviral response at non-cytotoxic concentrations of 1.25, 0.62, and 0.31 μM. (Fig. 1D–E), suggesting that the antiviral effect is mechanistically distinct from its cytotoxic activity. Moreover, viability assays conducted on both infected and uninfected HCE cells treated with doxorubicin revealed no significant cytotoxicity, further supporting the hypothesis that its antiviral effect is not attributable to its known cellular toxicity.

To further assess the therapeutic window, we calculated the selectivity index (SI) across a panel of immortalized and primary cell lines (Fig. 1A). Doxorubicin was consistently well tolerated at concentrations that robustly inhibited HSV-1 replication, confirming its broad-spectrum antiviral potential and further distinguishing its antiviral mechanism from its anticancer effects. This comparative approach allowed us to further delineate the antiviral properties of doxorubicin from its anticancer effects by evaluating its efficacy in both non-cancerous and cancerous cellular contexts. Based on these findings, we selected two representative models for further study: HCE cells, as a physiologically relevant model for HSV-1 infection, and HeLa cells, to examine antiviral efficacy in a cervical cancer cell line. HeLa cells infected with HSV-1 KOS were treated with doxorubicin, alongside ACV and TFT as controls. Doxorubicin treatment led to a dose-dependent, multi-log-fold reduction in viral titers, with antiviral efficacy comparable to standard antivirals despite being used at concentrations 40–80-fold lower (Fig. 1D). These observations were corroborated by qPCR and Western blot analyses, demonstrating a dose-dependent downregulation of key HSV-1 genes and proteins, incorporating ICP4, gB, and VP16, in both HCE and HeLa cells (Fig. 1E–G).

To evaluate the spectrum of antiviral activity, we extended the study to multiple HSV-1 strains, including multiple laboratory strains and clinical isolates, infecting HCE cells at an MOI of 0.1. Doxorubicin treatment consistently resulted in a dose-dependent, multi-log-fold reduction in both extracellular and intracellular viral titers across all the strains. Further, we tested the robustness of this antiviral effect under higher viral burden, we increased the MOI to 1 in HCE cells. Even under these conditions, doxorubicin treatment resulted in a substantial and statistically significant reduction in viral titers, again at concentrations much lower than those essential for ACV and TFT (Fig. 1H–I).

Due to the increasing interest in combining doxorubicin with oncolytic HSV-1 for cancer therapy, we examined whether doxorubicin could inhibit the replication of an oncolytic HSV-1 strain under *in vitro* conditions. Remarkably, doxorubicin demonstrated strong antiviral activity against the oncolytic virus, as shown by significant reductions in the expression of key viral transcripts, including ICP4, VP16, and gB (Fig. 1J). This transcriptional suppression was corroborated by plaque assays conducted at both low (0.1 MOI) and high (1.0 MOI) virus concentration, which revealed a dose-dependent, multi-log-fold reduction in viral titers (Fig. 1K). These findings underscore the broad-spectrum antiviral potential of doxorubicin across laboratory, clinical, and oncolytic HSV-1 strains and foster important considerations regarding its use in combination with oncolytic virotherapy.

### 2.2. Therapeutic application of doxorubicin suppresses infection by ACV-resistant clinical HSV-1 strains

Resistance to ACV and other nucleoside analogs has emerged after decades of antiviral therapy targeting a single viral mechanism, posing a major clinical challenge, especially in immunocompromised patients (Růžena et al., 2005). These challenges push forward a pressing need for the development of new antiviral agents. In this context, repurposing FDA-approved drugs offers a strategic and efficient approach. Therefore, we further evaluated its efficacy against clinically isolated ACV-resistant HSV-1 strains.

**Fig. 2.**
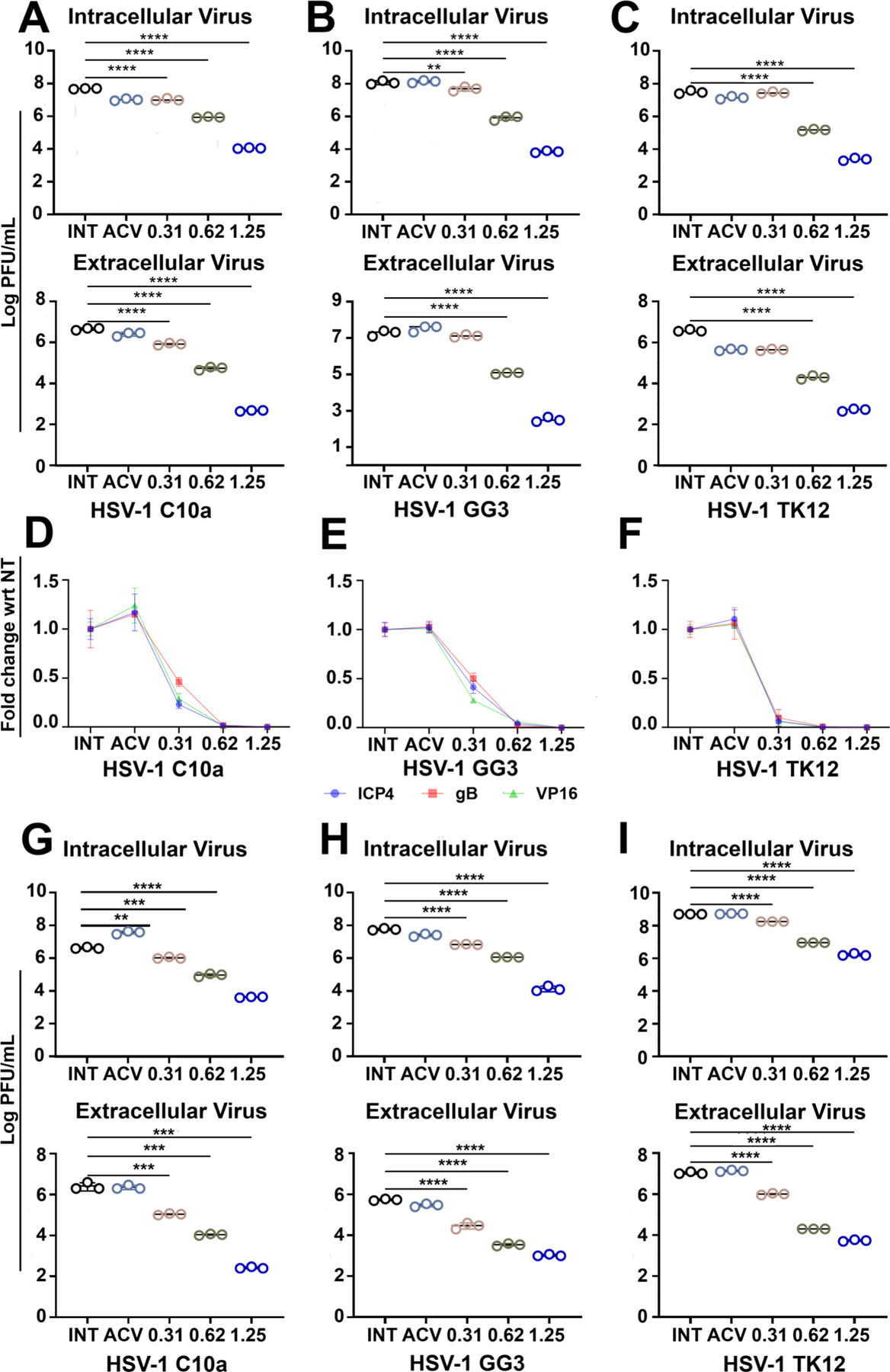
Doxorubicin suppresses acyclovir-resistant HSV-1 strains. (A–C) HCE cells were infected with clinically isolated acyclovir-resistant HSV-1 strains (GG3, C10a, and TK12) at a multiplicity of infection (MOI) of 0.1 and treated with doxorubicin (0.31–1.25 μM), acyclovir (ACV; 50 μM), or trifluridine (TFT; 50 μM). Intracellular and extracellular viral titers were measured by plaque assays at 24 h post-infection. (D–F) Quantitative PCR (qPCR) analysis showing doxorubicin-mediated reduction in viral transcripts (ICP4, VP16, and gB) in HCE cells infected with the indicated acyclovir-resistant HSV-1 strains. All doxorubicin treatments showed **** significance (p < 0.0001) by one-way ANOVA compared to the infected non-treated (INT) group, whereas ACV-treated samples were not significantly different from the INT group. (G–I) Dose-dependent, multi-log-fold reduction in intracellular and extracellular viral titers following doxorubicin treatment in HeLa cells infected with the same acyclovir-resistant HSV-1 strains (GG3, C10a, and TK12). All experiments were performed in three independent biological replicates (n = 3). Statistical analysis was conducted using one-way ANOVA.

HCE cells infected with these resistant strains were treated with doxorubicin at varying concentrations. In all cases, doxorubicin resulted in a consistent, dose-dependent, multi-log-fold reduction in both extracellular and intracellular viral titers. At 1.25 μM, doxorubicin demonstrated excellent antiviral efficacy, whereas ACV treatment, as expected, failed to reduce viral loads (Fig. 2A–2C). Further, qPCR analysis also confirmed a significant decrease in HSV-1 transcripts, including ICP4, gB, and VP16, following doxorubicin treatment (Fig. 2D–F). To assess whether these findings were cell-type specific, similar experiments were performed on HeLa cells, yielding comparable antiviral outcomes (Fig. 2G–I).

Collectively, these results demonstrate that doxorubicin maintains potent antiviral activity against ACV-resistant HSV-1 strains across different cellular contexts, further supporting its therapeutic potential and mechanistic distinction from traditional cytotoxicity.

### 2.3. Topical doxorubicin suppresses corneal HSV-1 infection in vivo without inducing local or systemic toxicity

Having established the antiviral efficacy of doxorubicin *in vitro* across multiple HSV-1 strains and diverse cell lines, we next evaluated its potential as a topical antiviral agent *in vivo* using a murine model of ocular HSV-1 infection. We assessed the antiviral efficacy of topically applied doxorubicin in comparison with mock-treated controls (DMSO) and trifluridine (TFT), the sole FDA-approved topical antiviral agent for ocular infections.

**Fig. 3.**
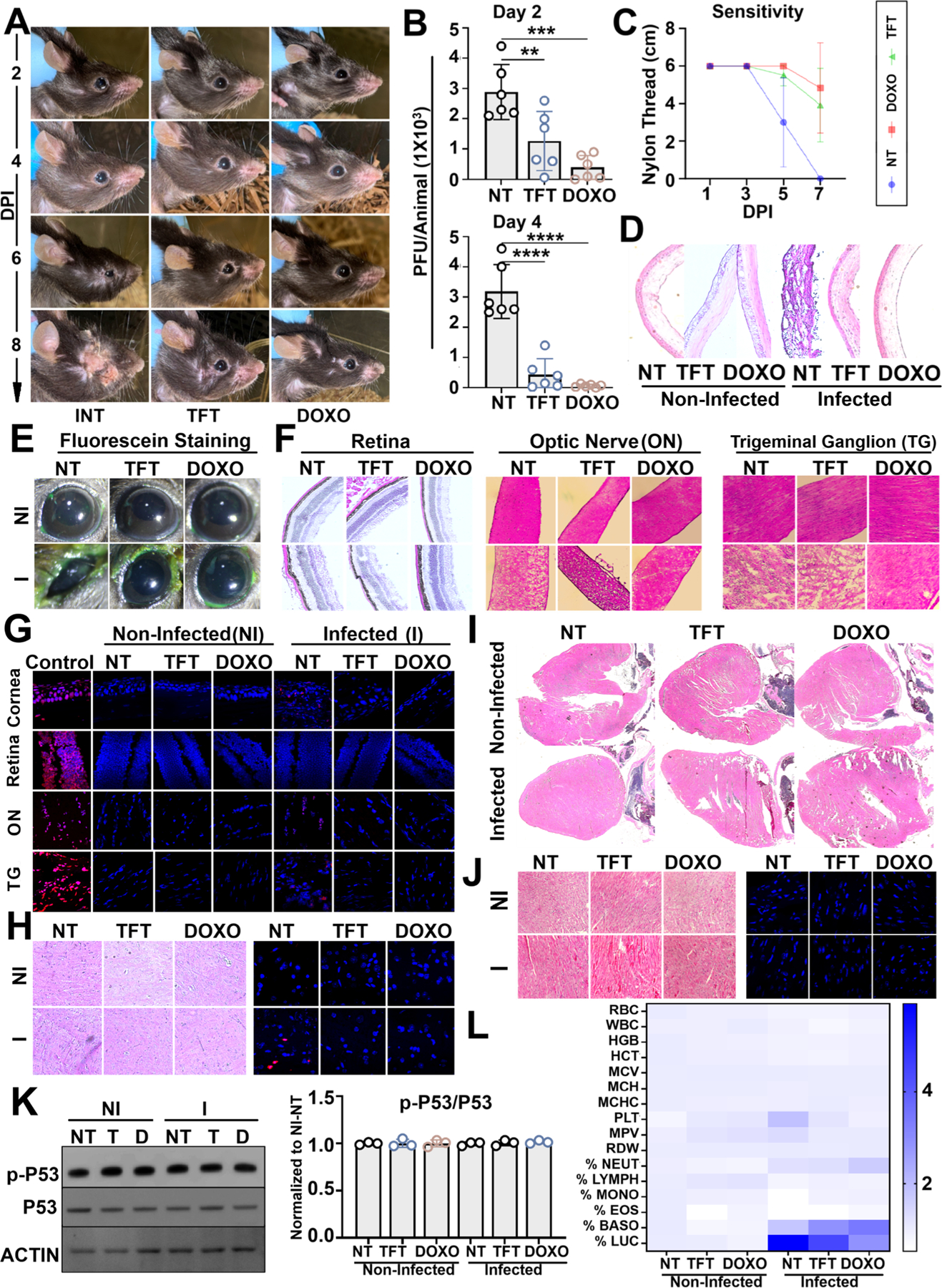
Topical application of doxorubicin attenuates corneal HSV-1 infection *in vivo*. (A) Representative images illustrating the progression of corneal pathology in mice infected with HSV-1 (McKrae strain) and treated with vehicle control (DMSO), trifluridine (TFT), or doxorubicin at the indicated days post-infection (DPI). (B) Plaque assays performed on eye-wash samples collected at 2 and 4 DPI demonstrating significantly reduced viral titers in TFT- and doxorubicin-treated groups compared with mock-treated controls. (C) Corneal sensitivity measurements across treatment groups at the indicated time points post-infection, indicating preservation of corneal function in drug-treated animals. (D) Hematoxylin and eosin (H&E) staining of corneal sections harvested at 8 DPI from non-treated (NT), TFT-treated, and doxorubicin-treated mice under non-infected and HSV-1-infected conditions, highlighting differences in inflammatory cell infiltration and tissue integrity. (E) Fluorescence staining demonstrating that topical doxorubicin treatment does not induce corneal surface toxicity at the working concentration. (F) H&E staining of retina, optic nerve (ON), and trigeminal ganglion (TG) showing doxorubicin-mediated protection against HSV-1–induced pathology. (G) TUNEL staining of cornea, retina, optic nerve, and trigeminal ganglion indicating absence of doxorubicin-associated tissue toxicity. (H) H&E staining of brainstem sections demonstrating protection against HSV-1 associated pathology (left), with corresponding TUNEL staining confirming absence of doxorubicin-induced toxicity at the tested concentration (right). (I) H&E staining of cardiac tissue showing that topical ocular administration of doxorubicin does not induce cardiac pathology. (J) Masson’s Trichrome X staining of cardiac tissue demonstrating absence of fibrosis following topical doxorubicin treatment (left), with accompanying TUNEL staining confirming lack of cardiomyocyte apoptosis (right). (K) Immunoblot analysis of p53 expression in mouse heart tissue from non-treated (NT), TFT-treated (T), and doxorubicin-treated (Doxo) groups (left), with corresponding densitometric quantification (right). (L) Complete blood count (CBC) analysis of mice receiving different treatments demonstrating absence of hematological toxicity or myelosuppression following topical doxorubicin administration. All animal experiments were performed using five mice per group (n = 5) and independently repeated at least three times. Statistical analysis was conducted using one-way ANOVA.

Mice were infected in the right eye with HSV-1 (McKrae). Treatment began at 1-day post-infection (DPI), with topical administration of doxorubicin (10 μM), trifluridine (TFT; 1 % solution, ~34 mM), or DMSO (vehicle control) three times daily. Eye wash samples collected revealed a significant reduction in viral titers in both doxorubicin- and TFT-treated groups compared to controls. Notably, doxorubicin, despite being applied at a nearly 3000-fold lower concentration than TFT, achieved comparable antiviral efficacy. Furthermore, mock-treated mice exhibited a complete loss of corneal sensitivity by 7 DPI, whereas corneal sensitivity was preserved throughout the treatment course in both TFT and doxorubicin-treated groups, indicating better maintenance of corneal integrity and reduced disease severity (Fig. 3A–C). To further assess ocular pathology, hematoxylin and eosin (H&E) staining of cornea at 8 DPI revealed marked inflammatory cell infiltration and tissue disruption in the mock-treated group. In contrast, eyes from doxorubicin- and TFT-treated mice exhibited reduced immune cell infiltration and lower levels of inflammation, consistent with effective suppression of viral replication and mitigation of HSV-1–associated immunopathology (Fig. 3D). To evaluate the antiviral efficacy of doxorubicin against oncolytic HSV-1, a parallel *in vivo* experiment was conducted in which mice were infected with an oncolytic HSV-1 strain in the right eye and treated topically with either doxorubicin or TFT. Eye wash samples demonstrated a similar pattern of viral suppression, with both treatments showing significant and dose-consistent reductions in viral titers, confirming the broad antiviral activity of doxorubicin even against genetically modified oncolytic HSV-1 strains (Fig. S1).

To evaluate local ocular toxicity, fluorescein staining was performed following topical doxorubicin administration and revealed no evidence of corneal surface damage at the working concentration (Fig. 3E). Given that ocular HSV-1 infection can spread to adjacent tissues, we next examined retina, optic nerve, trigeminal ganglion, and brainstem by H&E staining. These analyses showed reduced HSV-associated pathology in doxorubicin-treated animals, with no detectable drug-induced tissue damage (Fig. 3F). Because doxorubicin is a cytotoxic chemotherapeutic agent, we next assessed whether its antiviral activity was associated with induction of cell death. TUNEL staining of cornea, retina, optic nerve, trigeminal ganglion, and brainstem revealed no apoptotic cells in doxorubicin-treated animals, whereas infected mock-treated mice exhibited detectable TUNEL positivity, consistent with infection-associated cell death (Fig. 3G–H).

Given the known cardiotoxicity of systemically administered doxorubicin, we further evaluated potential cardiac toxicity following topical ocular treatment. H&E staining of heart sections revealed no pathological abnormalities across treatment groups, and Masson’s Trichrome X staining showed no evidence of cardiac fibrosis. As an additional proof-of-concept safety assessment, TUNEL staining of cardiac tissue demonstrated an absence of apoptotic cardiomyocytes (Fig. 3I–J). Moreover, immunoblot analysis of total p53 and phospho-p53 in heart tissue revealed no significant differences between treatment groups, indicating lack of doxorubicin-induced cardiac stress at the antiviral dose used (Fig. 3K).

Finally, to assess potential myelosuppression, complete blood count (CBC) analysis was performed. No significant alterations were observed in hematological parameters in doxorubicin-treated mice compared with controls, indicating absence of systemic hematological toxicity. Collectively, these data demonstrate that topical ocular administration of doxorubicin potently suppresses HSV-1 infection and associated pathology while remaining well tolerated, without inducing local tissue damage or systemic toxicity (Fig. 3L). These findings further support that the antiviral activity of doxorubicin at the concentrations used is mechanistically distinct from its anticancer cytotoxic effects.

### 2.4. Doxorubicin synergizes with nucleoside analogs to enhance antiviral efficacy and reduce effective drug concentrations

**Fig. 4.**
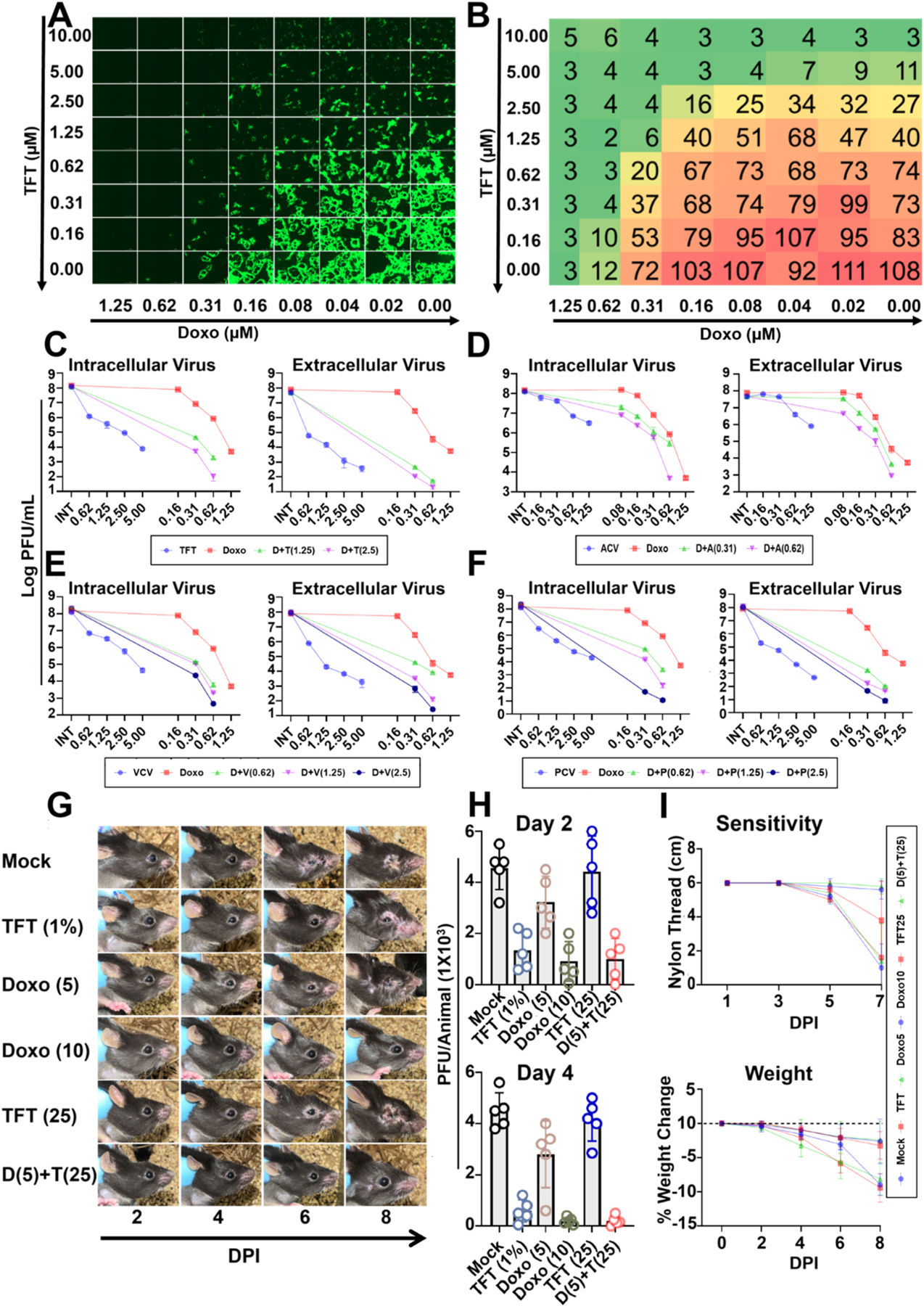
Doxorubicin synergizes with nucleoside analogs and reduces their effective antiviral concentrations. (A) HCE cells infected with HSV-1 K-26 GFP at 0.1 MOI were treated with trifluridine (TFT) and doxorubicin at varying concentrations individually and in combination using an 8 × 8 checkerboard format to assess synergy. (B) Quantification of GFP fluorescence intensity from (A) using a plate reader at 24 h post-infection. (C) Plaque assays confirming synergistic antiviral effects of doxorubicin and TFT combinations, showing significant reductions in intracellular and extracellular viral titers compared to individual treatments in HCE cells infected with HSV-1 McKrae. (D–F) Synergistic interactions between doxorubicin and acyclovir, valacyclovir, and penciclovir, demonstrating enhanced reductions in intracellular and extracellular viral titers with combinatorial therapy compared to individual drug treatments. (G) Representative images of mouse eyes collected at various days post-infection, following topical treatments with mock (DMSO), working concentrations of TFT (1 %) and doxorubicin (10 μM), non-working concentrations of TFT (25 μM) and doxorubicin (5 μM), and the combination of suboptimal TFT and doxorubicin (25 μM + 5 μM), showing enhanced therapeutic effects with combinatorial therapy. (H) Plaque assays quantifying viral titers from eye swabs collected at 2 and 4 DPI from treatment groups described in (G). (I–J) Assessment of corneal sensitivity and changes in body weight over time in mice receiving treatments described in (G). All animal experiments were performed with five mice per group (n = 5), and cell culture experiments were conducted with three independent biological replicates (n = 3).

Having established the antiviral properties of doxorubicin, we evaluated its potential to synergize with nucleoside analogs. Drug synergy describes a pharmacological interaction wherein the combined effect of two agents exceeds the additive effects of each agent alone. HCE cells infected with HSV-1 K26-GFP were treated with various concentrations of doxorubicin and TFT. Individually, both agents exhibited antiviral activity at higher concentrations; however, co-treatment revealed a pronounced synergistic effect at lower doses (Fig. 4A–B). Specifically, combinations of doxorubicin with TFT consistently achieved significant reductions in intracellular viral titers that could not be obtained by either drug alone at those concentrations.

Plaque assay analyses demonstrated that the combination of 0.31 μM doxorubicin with 1.25 μM TFT resulted in approximately a 10-fold greater reduction in viral titers compared to TFT alone and a ~175-fold greater reduction compared to doxorubicin alone. Increasing the TFT concentration to 2.5 μM with 0.31 μM doxorubicin further enhanced antiviral efficacy, achieving reductions of approximately 20-fold and 1500-fold relative to TFT and doxorubicin monotherapies, respectively. Similarly, the combination of 0.62 μM doxorubicin with 1.25 μM TFT led to ~400-fold and ~200-fold reductions compared to each monotherapy, while the combination of 0.62 μM doxorubicin with 2.5 μM TFT achieved a striking 3-log-fold and 4-log-fold reduction compared to TFT and doxorubicin alone, respectively (Fig. 4C). Notably, plaque numbers were markedly lower in all combination treatments compared to individual treatments. These synergistic effects were also observed for extracellular viral titers, further validating the combinatorial strategy.

To determine whether this synergy extended beyond TFT, we tested doxorubicin in combination with additional nucleoside analogs, including ACV and its prodrugs penciclovir and valacyclovir (Fig. S2). In each case, doxorubicin displayed consistent synergistic antiviral activity, producing substantial reductions in both intracellular and extracellular viral titers (Fig. 4D–F).

Encouraged by these findings, we next assessed the therapeutic potential of a doxorubicin and TFT combination in a murine model of ocular HSV-1 infection. Co-administration of suboptimal concentrations of doxorubicin (5 μM) and TFT (25 μM) resulted in a substantial reduction in viral titers, effectively halving the required therapeutic dose of TFT without compromising efficacy. Eye wash samples collected post-infection revealed a significant reduction in plaque-forming units in the combination-treated group, whereas corresponding monotherapies at these suboptimal doses failed to reduce viral titers or alleviate disease severity. Moreover, mice receiving the combinatorial therapy retained corneal sensitivity throughout the course of infection, in contrast to mock-treated animals and those receiving monotherapy, which exhibited complete loss of corneal sensitivity (Fig. 4G–I).

### 2.5. Therapeutic application of doxorubicin effectively curbs HSV-1 infection in a porcine ex vivo corneal model and primary porcine corneal cells

**Fig. 5.**
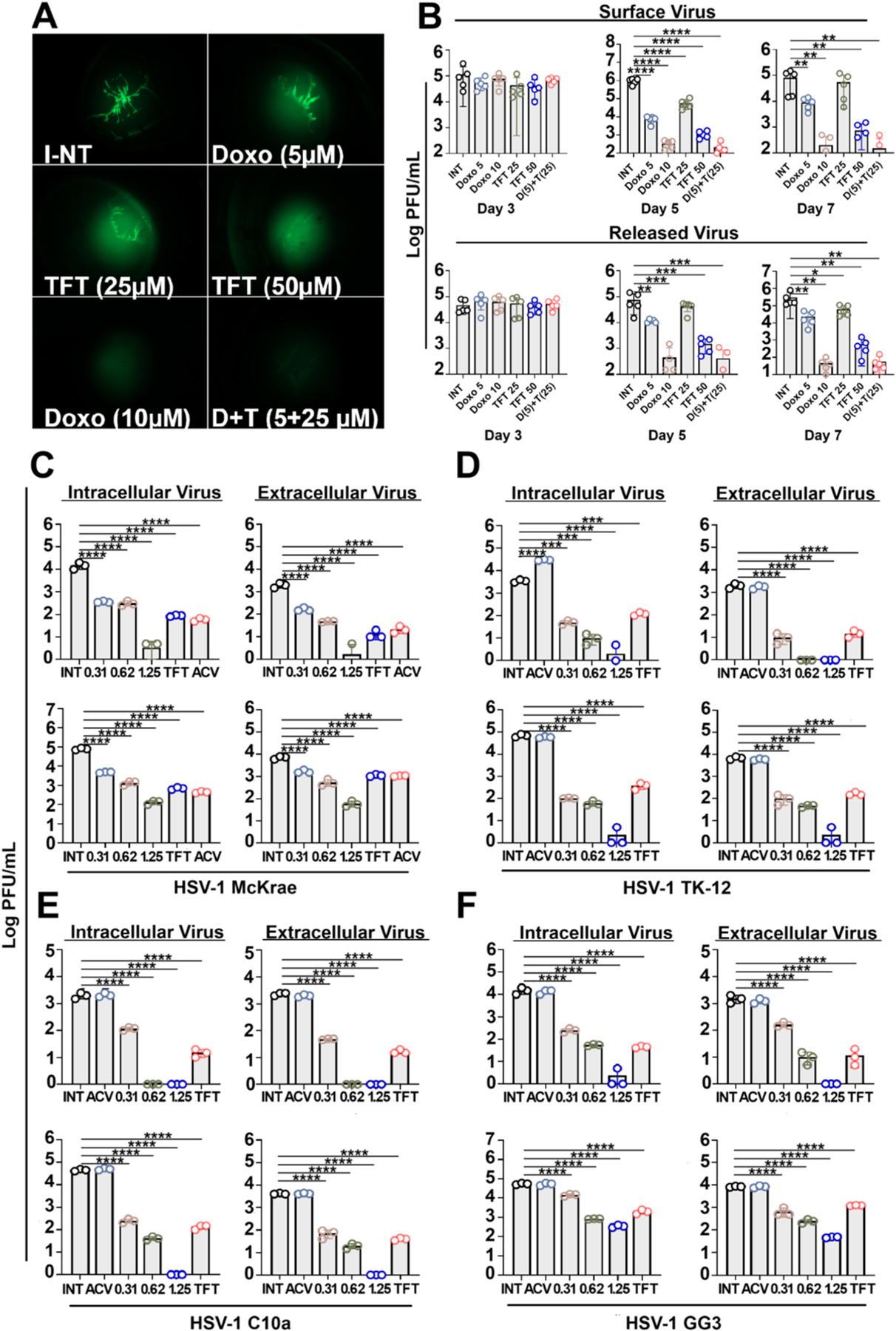
Doxorubicin exhibits potent antiviral efficacy in a porcine ex vivo corneal model and in primary porcine corneal cells. (A) Representative images of porcine corneas at 7 days post-infection (DPI) with HSV-1 17-GFP, following topical treatment with mock (DMSO), doxorubicin (5 or 10 μM), trifluridine (TFT; 25 or 50 μM), or a combination of suboptimal concentrations of doxorubicin and TFT (5 μM + 25 μM). (B) Quantification of surface-associated and released viral titers by plaque assay at 3, 5, and 7 DPI for the treatment groups described in (A). (C) Plaque assay quantification demonstrating significant reductions in intracellular and extracellular viral titers in primary porcine corneal fibroblasts infected with HSV-1 McKrae at low (0.1 MOI; top panel) and high (1.0 MOI; bottom panel) infection doses, following doxorubicin treatment. (D–F) Doxorubicin-mediated reduction in intracellular and extracellular viral titers in primary porcine corneal fibroblasts infected with clinically isolated acyclovir-resistant HSV-1 strains (TK12, C10a, and GG3) at both low (0.1 MOI; top panels) and high (1.0 MOI; bottom panels) infection doses. All *ex vivo* infection experiments were performed using five independent porcine corneal samples per group (n = 5), and all primary cell culture experiments were conducted with three independent biological replicates (n = 3). Statistical analyses were performed using one-way ANOVA, with comparisons made against the infected non-treated (INT) group.

To further verify the therapeutic efficacy and universality of doxorubicin’s antiviral effect fresh porcine eyes were obtained from a local abattoir, and corneas were excised using a previously established protocol with minor modifications (Yadavalli et al., 2021). The eyes were infected with HSV-1(17-GFP) and topical treatments were administered. We observed a significant reduction in both surface-associated and released viral titers in corneas treated with 10 μM doxorubicin and 50 μM trifluridine (TFT). While treatment with suboptimal concentrations of either doxorubicin or TFT alone led to a modest reduction in viral load, the combination of 5 μM doxorubicin and 25 μM TFT produced the most pronounced antiviral effect, resulting in a multi-log reduction in viral titers (Fig. 5A–B).

To further dissect the antiviral effects of doxorubicin, primary cells were isolated from porcine corneas and used to evaluate viral replication kinetics upon treatment. These cells were infected with low or high doses of both ACV-sensitive and -resistant HSV-1 strains, followed by treatment with doxorubicin, TFT, or ACV. Compared to immortalized lines, primary pig corneal cells exhibited increased resistance to infection, with viral titers ranging between 10^4^ PFU (0.1 MOI) and 10^5^ PFU (1.0 MOI) in untreated controls. A clear dose-dependent antiviral response to doxorubicin was observed, with complete inhibition (no detectable plaques) at 1.25 μM under low MOI conditions. Even under high MOI infection, doxorubicin significantly reduced viral titers, outperforming standard antivirals used at much higher concentrations (50 μM). Importantly, doxorubicin retained robust antiviral activity against multiple ACV-resistant clinical isolates across both MOI conditions, mirroring its effectiveness observed earlier (Fig. 5C–F).

### 2.6. Doxorubicin’s antiviral activity is mediated through inhibition of the PI3K-AKT pathway

**Fig. 6.**
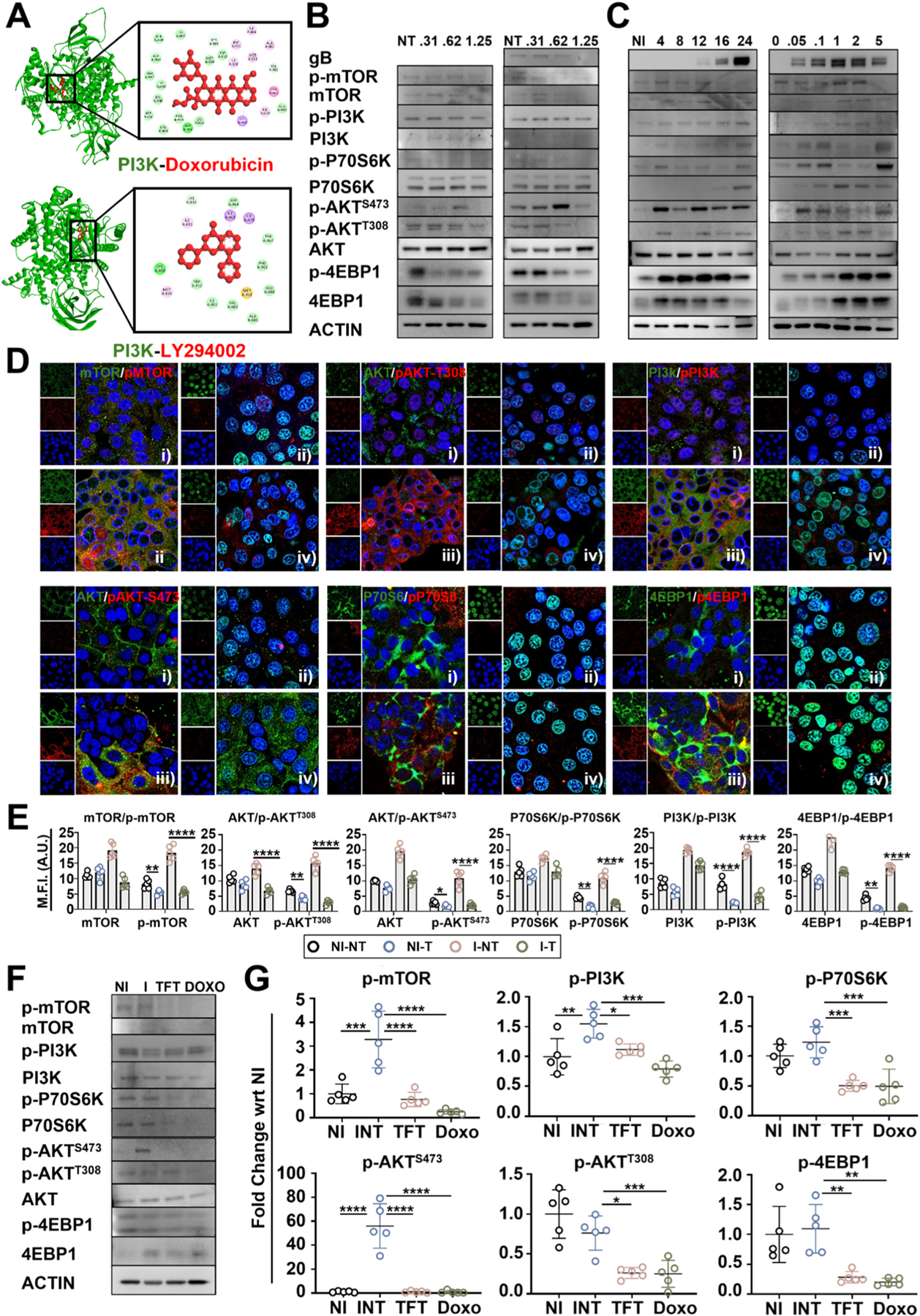
Doxorubicin inhibits HSV-1 infection by blocking the PI3K–AKT–mTOR signaling pathway. (A) *In-silico* molecular docking analysis illustrating the interaction between doxorubicin (ball-and-stick representation) and PI3K (cartoon representation), performed using Discovery Studio. (B) Immunoblot analysis of HCE cells treated with increasing concentrations of doxorubicin, showing inhibition of PI3K and downregulation of downstream signaling molecules (AKT, mTOR, p70S6K, and 4EBP1) under non-infected (left) and HSV-1 McKrae-infected (right; MOI 0.1) conditions. (C) Immunoblot analysis demonstrating HSV-1–induced activation of the PI3K–AKT–mTOR pathway in HCE cells in a time-dependent (left panel) and MOI-dependent (right panel) manner. (D) Immunofluorescence staining of HCE cells showing reduced expression of PI3K, phospho-AKT, and phospho-mTOR following doxorubicin treatment. (E) Quantification of mean fluorescence intensity from (D), performed using ImageJ software. (F) Immunoblot analysis of PI3K pathway markers in corneal tissues isolated from HSV-1–infected mice treated with vehicle (mock), doxorubicin, or trifluridine (TFT). (G) Densitometric quantification of immunoblot data shown in (F), normalized to β-actin expression. Statistical analyses were performed using one-way ANOVA in GraphPad Prism; significance levels are indicated in the respective figure panels.

Following the characterization of doxorubicin’s antiviral efficacy against multiple HSV-1 strains across various cell lines, we investigated the underlying mechanism responsible for its activity. We initially performed *in-silico* docking studies and identified phosphoinositide 3-kinase (PI3K) as a potent target, suggesting strong binding affinity (−9.7 kcal/mol) compared to known PI3K inhibitors including Pictilisib, Alpelisib, Taselisib, Copanlisib, Wortmannin, and LY294002, as positive controls, with ACV serving as a negative control. The respective binding affinities (in kcal/mol) of these inhibitors were −9.1, −7.8, −8.3, −8.2, −8.4, and −9.6 (Fig. 6 A and S3).

To experimentally validate the predicted inhibition of PI3K signaling, we performed Western blot analysis in HCE cells. Doxorubicin treatment led to a marked downregulation of PI3K expression, accompanied by reduced phosphorylation of downstream effectors, including AKT, P70S6K, mTOR, and 4EBP1 (Fig. 6B). In contrast, HSV-1 infection resulted in a significant upregulation of these signaling components, with both time-dependent and dose-dependent increases observed, consistent with activation of the PI3K-AKT-mTOR pathway during infection (Fig. 6C). To further support our findings, we performed immunofluorescence staining in HCE cells targeting key components of the PI3K-AKT-mTOR signaling pathway. As expected, HSV-1 infection led to a substantial upregulation of all markers within this signaling axis, corroborating our previous observations from western blot analyses. Notably, doxorubicin treatment resulted in a marked downregulation of these markers, even in the absence of infection, as evidenced by significantly reduced mean fluorescence intensity (MFI) in the mock-infected/doxorubicin-treated group compared to mock-infected/mock-treated controls. The effect was even more pronounced in the HSV-1-infected/doxorubicin-treated group, which exhibited a multifold reduction in MFI relative to HSV-1-infected/mock-treated cells (Fig. 6D–E). To establish the functional requirement of PI3K-AKT signaling for HSV replication, we next performed pharmacological gain- and loss-of-function experiments. Inhibition of PI3K using LY294002 significantly reduced viral replication, whereas pharmacological activation of AKT using SC79 enhanced HSV-1 infection (Fig. S4D–E) These results provide direct functional evidence that PI3K-AKT signaling positively regulates HSV-1 replication and that suppression of this pathway restricts infection. Given the growing clinical relevance of oncolytic herpesviruses, we next examined whether oncolytic HSV-1 similarly depends on PI3K-AKT signaling. Immunoblot analyses revealed time and dose-dependent activation of the PI3K-AKT pathway during oncolytic HSV infection (Fig. S4A). Consistent with this observation, pharmacological inhibition of PI3K using LY294002 suppressed oncolytic HSV replication, whereas AKT activation using SC79 enhanced viral replication (Fig. S4B–C, indicating that productive replication of oncolytic HSV likewise requires PI3K-AKT pathway activation.

To disentangle PI3K-AKT-mediated antiviral effects from doxorubicin’s canonical topoisomerase II inhibition, we utilized a doxorubicin-resistant H69AR cell line, which is characterized by markedly reduced topoisomerase II expression. Importantly, doxorubicin treatment in H69AR cells retained the ability to suppress PI3K-AKT signaling and significantly reduced viral load following infection with both wild-type HSV-1 and oncolytic HSV-1 (Fig. S4F), demonstrating that doxorubicin’s antiviral activity is independent of topoisomerase II inhibition. Consistent with this conclusion, immunoblot analysis of phospho-γH_2_AX, a marker of DNA damage and topoisomerase II poisoning, revealed no induction and instead a reduction of γH_2_AX phosphorylation at antiviral concentrations of doxorubicin (Fig. S4F), indicating absence of a genotoxic stress response.

To evaluate the physiological relevance of these findings, we extended our investigation to an *in vivo* murine model of ocular HSV-1 infection. As expected, the infected and mock-treated group exhibited significant upregulation of the PI3K-AKT-4EBP1 signaling pathway, consistent with previous reports describing HSV-1–mediated exploitation of this host axis. In contrast, eyes from doxorubicin-treated mice displayed a pronounced downregulation of this pathway, supporting our *in-silico* and *in vitro* findings (Fig. 6F–G). Mice treated with TFT also exhibited reduced expression of these markers, likely due to lower viral loads, suggesting an indirect effect rather than direct pathway inhibition. Collectively, these findings demonstrate that PI3K-AKT signaling is a conserved host dependency for productive replication of both wild-type and oncolytic HSV, and that doxorubicin exerts its antiviral activity predominantly through host-directed inhibition of this pathway, rather than through genotoxic or topoisomerase II-dependent mechanisms.

### 2.7. Pre-treatment with doxorubicin inhibits HSV-1 entry and replication in vitro

**Fig. 7.**
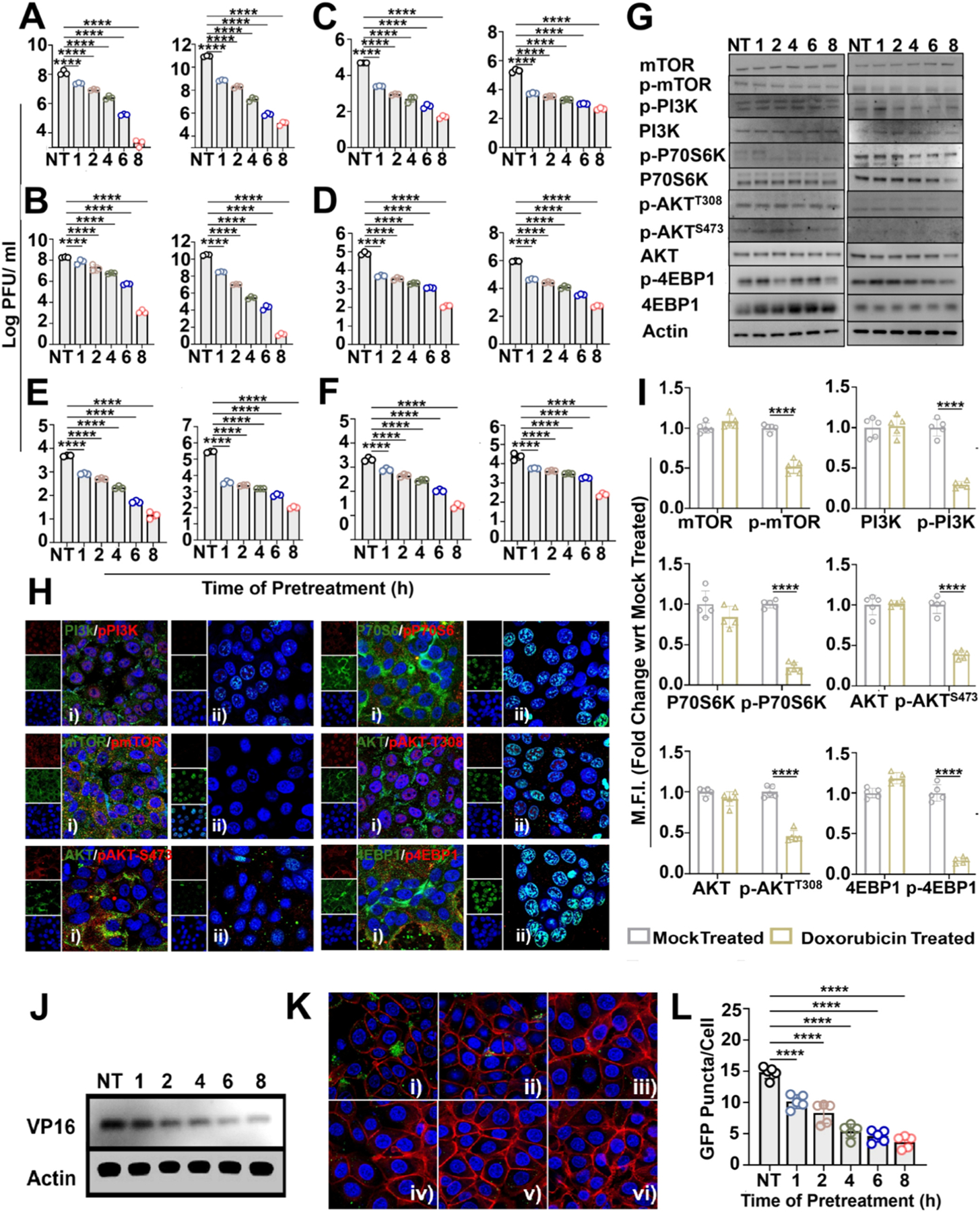
Pretreatment with doxorubicin inhibits HSV-1 infection and viral entry. (A, B) HCE cells were pretreated with 1.25 μM doxorubicin for increasing durations (1–8 h) prior to infection with HSV-1 McKrae. Intracellular (A) and extracellular (B) viral titers were quantified by plaque assays at low (0.1 MOI; left) and high (1.0 MOI; right) infection conditions. (C, D) Primary murine dermal fibroblasts were pretreated with doxorubicin and subsequently infected with HSV-1 McKrae. Intracellular (C) and extracellular (D) viral titers were determined by plaque assays at 0.1 (left) and 1.0 MOI (right). (E, F) Primary porcine corneal fibroblasts were pretreated with doxorubicin for increasing durations and then infected with HSV-1 McKrae. Intracellular (E) and extracellular (F) viral titers were measured by plaque assays at both 0.1 (left) and 1.0 MOI (right). (G) Western blot analysis showing time-dependent downregulation of PI3K–AKT–mTOR pathway components following doxorubicin pretreatment in HCE cells (left) and primary murine dermal fibroblasts (right). (H) Immunofluorescence staining of total and phosphorylated forms of PI3K, AKT, mTOR, p70S6K, and 4EBP1 in HCE cells pretreated with doxorubicin for 8 h. (I) Quantification of mean fluorescence intensity from (H), analyzed using ImageJ. (J) Western blot analysis showing reduced expression of HSV-1 entry protein VP16 in HCE cells pretreated with doxorubicin, followed by infection at 20 MOI. (K) Confocal microscopy images showing decreased intracellular accumulation of GFP-tagged HSV-1 (K26-GFP) in HCE cells pretreated with doxorubicin for 1 h (ii), 2 h (iii), 4 h (iv), 6 h (v), and 8 h (vi) as compared to non-treated (i). (L) Quantification of intracellular GFP-positive puncta per cell from images in (K), analyzed using ImageJ. Statistical comparison to the non-treated (NT) group was performed using one-way ANOVA. All plaque assay experiments were performed in triplicate (n = 3) and analyzed using one-way ANOVA. Quantification of immunofluorescence intensity was assessed using unpaired *t*-tests.

PI3K activation is known to occur early during HSV-1 infection and plays a critical role in facilitating viral entry. Given our earlier findings on the PI3K-inhibitory potential of doxorubicin, we investigated whether pre-treatment with doxorubicin could suppress HSV-1 infection *in vitro*. HCE cells pre-treated with doxorubicin were infected with HSV-1 McKrae followed by the assessment of extracellular and intracellular viral titers. We observed a significant decline in the viral titer corresponding to the time of pre-treatment, resulting in nearly 5-log reduction in viral load with a pretreatment of 8 h (Fig. 7A–B). To determine whether this effect was preserved at higher viral loads, we repeated the experiment using a multiplicity of infection (MOI) of 1, and observed a similar trend, confirming that doxorubicin pre-treatment inhibits both low and high-dose HSV-1 infections. These findings were further validated in primary murine dermal fibroblasts and primary porcine corneal fibroblasts, where a similar time-dependent decline in both extracellular and intracellular viral titers was (Fig. 7C–F).

To further correlate the observed antiviral effect with modulation of the PI3K signaling pathway, we performed Western blot analyses on HCE cells and primary murine dermal fibroblasts pretreated with doxorubicin for varying durations. A progressive downregulation of PI3K and its downstream effectors including AKT, mTOR, and 4EBP1 was observed with increasing pretreatment time, indicating a time-dependent inhibition of the pathway (Fig. 7G). Complementary immunofluorescence analysis also revealed a significant reduction in PI3K pathway activation compared to the mock-treated group, as quantified by decreased mean fluorescence intensity (Fig. 7H–I).

Since PI3K signaling is known to facilitate viral entry, we next investigated whether the antiviral effects of doxorubicin were attributable to inhibition of viral entry. We quantified HSV-1 entry into HCE cells by measuring levels of the early viral protein VP16. In line with our previous observations, VP16 expression decreased in a time-dependent manner, correlating with increasing durations of doxorubicin pre-treatment, thereby confirming impaired viral entry. Using a confocal imaging approach with KOS-26-GFP virus, we further quantified internalized GFP-positive viral puncta. Doxorubicin pre-treatment significantly reduced the number of viral particles entering host cells in a time-dependent manner (Fig. 7J–L).

### 2.8. Prophylactic application of suboptimal doses of doxorubicin enhances the therapeutic efficacy of nucleoside analogs against HSV-1

Chemotherapy is a well-established trigger for HSV-1 reactivation, primarily due to the associated immunosuppressive effects. When immune surveillance is compromised, latent HSV-1 residing in sensory ganglia can reactivate, leading to recurrent infections and, in severe cases, herpes simplex encephalitis. To reduce this risk, prophylactic antiviral therapy is commonly recommended during and after chemotherapy, particularly in high-risk patients (Sandherr et al., 2006).

**Fig. 8.**
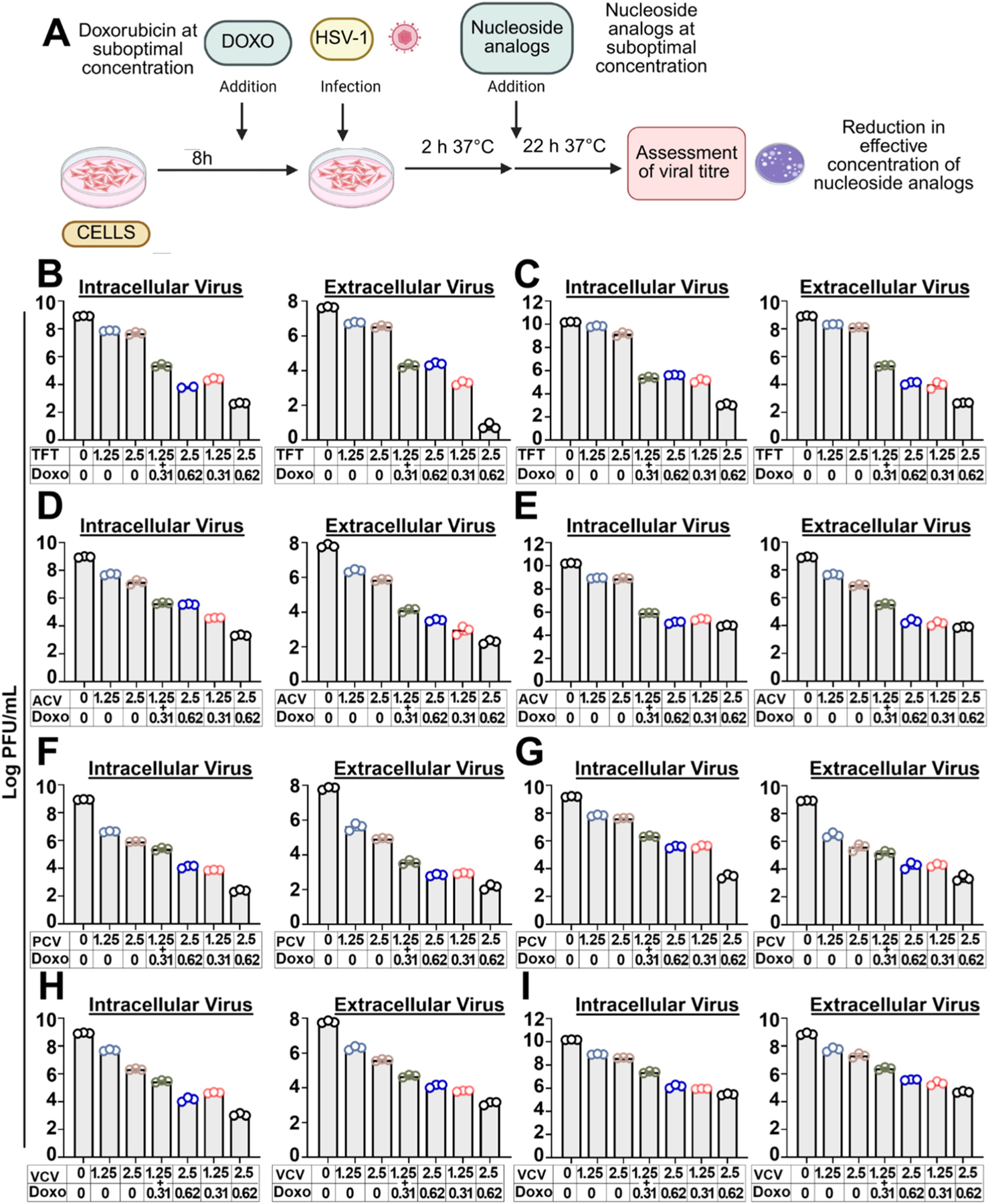
Pretreatment with suboptimal concentrations of doxorubicin enhances the therapeutic efficacy of nucleoside analogs. (A) Schematic representation of the experimental workflow illustrating doxorubicin pretreatment, HSV-1 infection, and subsequent treatment with nucleoside analogs. (B, C) Pretreatment with suboptimal concentrations of doxorubicin (0.31–0.62 μM) significantly enhances the antiviral efficacy of trifluridine (TFT), resulting in marked reductions in both intracellular and extracellular HSV-1 titers compared to TFT monotherapy at low (0.1 MOI; B) and high (1.0 MOI; C) infection conditions. (D, E) Prophylactic doxorubicin administration significantly improves the antiviral activity of acyclovir (ACV), as evidenced by reduced viral titers in HCE cells infected at 0.1 MOI (D) and 1.0 MOI (E). (F, G) Doxorubicin pretreatment potentiates the antiviral efficacy of penciclovir (PCV), leading to significant reductions in viral titers at both 0.1 MOI (F) and 1.0 MOI (G) compared to PCV monotherapy. (H, I) Similarly, doxorubicin pretreatment enhances the antiviral activity of valacyclovir (VCV), resulting in significantly lower HSV-1 titers than VCV alone at 0.1 MOI (H) and 1.0 MOI (I). All plaque assay experiments were performed in three independent biological replicates (*n* = 3). Statistical analyses were conducted using one-way ANOVA. Combination treatments exhibited **** significance (*p* < 0.0001) compared to the respective monotherapies.

To test the effects of prior treatment with doxorubicin on conventional antiviral treatment, HCE cells were pretreated with doxorubicin followed by HSV-1 infection (Fig. 8A). Cells that were pretreated with 0.31 μM doxorubicin and subsequently treated with TFT exhibited approximately a 300-fold and 8000-fold reduction in intracellular viral titers at 1.25 μM and 2.5 μM TFT, respectively, compared to cells that received TFT alone. Notably, pretreatment with 0.62 μM doxorubicin resulted in an even more pronounced antiviral effect, leading to approximately 3000-fold and 111,111-fold reductions in intracellular viral titers at 1.25 μM and 2.5 μM TFT, respectively. These results clearly demonstrate the synergistic enhancement of TFT efficacy following prophylactic administration of suboptimal concentrations of doxorubicin. A similar enhancement was also observed in extracellular viral titers, highlighting the additive effect of doxorubicin pretreatment in potentiating TFT’s therapeutic efficacy. This enhancement remained evident even under higher infection loads (1 MOI), further supporting the robustness of the effect (Fig. 8B–C).

Since TFT is limited to topical administration, we next evaluated whether ACV, a systemically administered standard-of-care antiviral that can cause nephrotoxicity with prolonged use, also benefits from doxorubicin pretreatment. HCE cells were pretreated with doxorubicin, followed by HSV-1 infection and therapeutic administration of ACV. We observed a ~1400-fold and ~7000-fold reduction in intracellular viral titers, respectively, in cells pretreated with 0.62 μM doxorubicin, as compared to those receiving ACV alone. Extracellular viral titers followed a similar trend, confirming the synergistic potential of the combination. To further validate our findings, we extended this strategy to ACV prodrugs, including penciclovir and valacyclovir, under both low (0.1 MOI) and high (1 MOI) infection conditions. In all cases, prophylactic doxorubicin pretreatment significantly enhanced the antiviral efficacy of these nucleoside analogs, in terms of both intracellular and extracellular viral load reduction (Fig. 8D–I).

Collectively, these results demonstrate that suboptimal concentrations of doxorubicin, when used as a prophylactic intervention, sensitize host cells to subsequent antiviral treatments, significantly lowering the effective therapeutic dose required for nucleoside analogs. This approach may hold clinical value, particularly in cancer patients undergoing chemotherapy who are at elevated risk for HSV-1 reactivation.

## 3. Discussion

HSV-1 is a highly prevalent human pathogen associated with a broad spectrum of clinical manifestations, many of which remain incompletely understood (Armangue et al., 2018; Antony et al., 2024). Despite its substantial global disease burden, therapeutic options for HSV-1 remain limited, with a focus on direct inhibition of viral DNA replication. Although nucleoside analogs and newer helicase-primase inhibitors, such as pritelivir and amenamevir, have improved clinical outcomes, these agents converge on a narrow set of viral targets, resulting in strong selective pressure for the emergence of drug-resistant variants, particularly in immunocompromised patients requiring prolonged or repeated treatment (Bacon et al., 2003). Compounding this challenge, current treatment paradigms largely lack mechanistically informed combinatorial strategies capable of enhancing antiviral efficacy while simultaneously suppressing resistance development.

These limitations underscore a critical unmet need for alternative therapeutic approaches that move beyond direct viral inhibition. In this context, host-directed antiviral strategies, particularly those amenable to rational combination with existing antivirals, represent an attractive and potentially resistance-resilient solution (Jaishankar et al., 2018; Wallin et al., 2010; Mertens and Knipe, 2021; Orvedahl et al., 2007). Importantly, the repurposing of clinically approved drugs such as doxorubicin to fulfill this role offers a uniquely rapid and practical translational pathway, leveraging established safety profiles to accelerate deployment while addressing both therapeutic efficacy and the growing problem of antiviral resistance. Through comprehensive, *in vitro*, *ex vivo*, and *in vivo* analyses, we demonstrate that doxorubicin robustly inhibits HSV-1 entry and replication by targeting the host PI3K-AKT-mTOR signaling axis. Doxorubicin exhibited broad-spectrum efficacy against multiple HSV-1 strains, including ACV-resistant clinical isolates, and synergized with nucleoside analogs to enhance antiviral potency while reducing effective drug doses. More specifically, our study identifies host-directed inhibition of the PI3K-AKT-mTOR pathway as a central mechanism underlying doxorubicin’s antiviral activity against HSV-1. In addition to harnessing drug repurposing as a strategy, our findings demonstrate that targeted suppression of a conserved host signaling axis required for viral entry and productive replication can effectively restrict HSV-1 infection, providing mechanistic and translational insights into resistance-refractory antiviral design.

The link between chemotherapy and HSV-1 reactivation is well established. Multiple chemotherapeutic classes including anthracyclines, alkylating agents (e.g., cyclophosphamide), purine analogs (e.g., fludarabine), anti-CD20 monoclonal antibodies (e.g., rituximab), and proteasome inhibitors (e.g., bortezomib) have been associated with an elevated risk of herpesvirus reactivation. Chemotherapy-induced immunosuppression impairs both innate and adaptive immune responses, facilitating viral reactivation from latency. Clinical evidence strongly supports this connection: Masci et al. reported herpes simplex and herpes zoster infections in patients undergoing chemotherapy (Masci et al., 2007). Similarly, Hong et al. demonstrated a correlation between oral HSV-1 reactivation and chemotherapy-induced mucositis (Hong et al., 2019). Patamatamkul et al. described a case of disseminated HSV-1 infection during post-chemotherapy neutropenia (Patamatamkul et al., 2024). Likewise, Saito et al. reported HSV-1 encephalitis in an esophageal cancer patient receiving chemoradiation and corticosteroids (Saito et al., 2016). Historical randomized trials also showed that HSV-1 reactivation occurred in up to 73 % of leukemia patients undergoing induction chemotherapy without antiviral prophylaxis, a risk significantly mitigated by ACV administration (Sandherr et al., 2006; Henze et al., 2022). Doxorubicin can inhibit topoisomerase II and forms adducts with DNA, both of which have the potential to interfere with HSV-1 replication (Cutts et al., 2005). Our study suggests that doxorubicin not only exhibits direct antiviral activity against HSV-1 but could also serve as a preferred chemotherapeutic agent for patients with a history of HSV-1 infection. Furthermore, administration of doxorubicin during chemotherapy may not only lower the incidence of HSV-1 reactivation but also enable a significant reduction in the effective doses of nucleoside analogs required for viral suppression post-chemotherapy. This dual benefit could ultimately enhance therapeutic outcomes while minimizing the toxicity associated with high-dose antiviral therapies.

Our study presents compelling evidence supporting the repurposing of doxorubicin as a novel antiviral agent. Drug repurposing has emerged as a powerful strategy for rapidly expanding antiviral treatment options by leveraging the established pharmacological and safety profiles of existing agents. Historically, several compounds originally developed for non-antiviral indications have been successfully redirected to treat high-morbidity viral infections. Zidovudine (AZT), initially synthesized as a thymidine analog for leukemia chemotherapy, became the first FDA-approved antiretroviral agent for HIV-1/AIDS. Repurposing doxorubicin could therefore offer a novel therapeutic avenue to mitigate HSV-1 recurrence, particularly in vulnerable populations undergoing chemotherapy, while simultaneously optimizing antiviral regimens through synergistic dose reduction strategies. The repurposing should be done with extra caution when combining doxorubicin with oncolytic herpesvirus therapy against cancer, as potential interactions may reduce cancer-targeting efficacy (Zheng et al., 2014; Zhuang et al., 2012).

In summary, our study identifies doxorubicin as a potent antiviral agent against HSV-1, distinct from its established anticancer activity. Our findings establish a strong translational rationale for repurposing doxorubicin as a novel adjunct antiviral strategy for HSV-1 infections, particularly in immunocompromised patients, and warrant further preclinical and clinical evaluation.

## 4. Materials & methods

### 4.1. Cells and virus

HeLa cells, H69AR cells and Vero cells were bought from ATCC. Vero cells were used in plaque assays and virus preparation. HCE cells were provided by Dr. Kozaburo Hayashi (National Eye Institute, Bethesda, MD). Mouse Embryonic Fibroblasts (MEFs) were provided by Dr. Israel Vlodavsky (Rappaport Institute, Haifa, Israel), Primary Pig Corneal Fibroblast (PCF) cells were isolated from Pig eyes provided by Peoria Packings, Chicago and Primary Mouse Adult Fibroblast (MAFs) cells were isolated from mice. All cells were cultured in Dulbecco’s Modified Eagle Medium (DMEM; # MT10013CV), except HCE cells, which were cultured in Minimum Essential Medium (MEM; # MT10010CV) and H69AR cells were cultured in RPMI 1640 (# MT10040CV; Mediatech, Inc.). The media was supplemented with 1 % penicillin/streptomycin (Mediatech, Inc; #MT30002CI) and 10 % fetal bovine serum (FBS; Sigma-Aldrich; #F2442–500mL).

Dr. Patricia Spear (Northwestern University) provided the KOS and F strains. Dr. Homayon Ghiasi (Cedars Sinai Medical Center) provided the McKrae, Oncolytic HSV-1 and 17 strains. Dr. Prashant Desai (Johns Hopkins University) provided the HSV-1 K26-GFP KOS strain. UL and BL strains (ULS and BLS) were isolated from ocular material of patients. ACV resistant strains (GG3, TK12 and C10a) were provided by Donald M. Coen (Harvard Medical School).

### 4.2. Mice

Eight- to ten-week-old C57/BL6 mice were used in the experiments. All experiments were performed and housed in a BSL2 rodent facility in the Biologic Resources Laboratory at the University of Illinois at Chicago with a standard 12 h light, 12 h dark cycle. Ambient temperatures at this facility are maintained between 20 and 26 °C and relative humidity between 45 % and 65 %. This modern animal facility has several veterinarians on staff available for expert veterinary care and advice during the project. Animal services core facility at the Department of Ophthalmology and visual sciences houses a BSL-2 facility dedicated specifically to our laboratory use. All protocols have been reviewed and certified by the animal care committee of the University of Illinois at Chicago.

### 4.3. Mouse infection experiments

On day 0, n = 5 (each group) mice were anesthetized with an intraperitoneal injection of ketamine (100 mg/kg) and xylazine (5 mg/kg). Afterward, they were given 0.5 % proparacaine hydrochloride topically. Corneal epithelial debridement in a 3 × 3 grid pattern was performed using a 30-gauge needle. A total of 1 × 10^4^ PFU HSV-1 McKrae viruses were overlaid on the debrided cornea in a total volume of 5 μL. Animals were monitored until awake and then allowed to return to their cages. Murine corneas were treated with doxorubicin (10 μM), TFT (1 %) and PBS as negative control respectively. Treatments were given as 5 μl eye drops 3 times every day for 5 days. On day 2 and 4 post infection, ocular washes were collected in DPBS for viral titration. Virus titration was performed on Vero Cells to quantify extent of infectious virus prevalent in murine eyes. Animal weights were monitored daily until the end of the experimental period.

### 4.4. Esthesiometry

Corneal sensitivity of the mice eyes was measured by manual Esthesiometer (12/100 mm, LUNEAU SAS, France). Corneal sensitivity measurements as a function of blink response were conducted on wake mice. The nylon fiber of the esthesiometer was extended to full length (6 cm), and the tip of the nylon thread was gently impressed to the center of the cornea. In a normal cornea, this should inflict a blink response which corresponds to a sensitivity score. If the mouse did not blink, the length of the nylon fiber was reduced by 0.5 cm, and the exercise was repeated until a blink response was observed. The results for this study were interpreted as follows: Score of 5.5–6 as normal, 5.5–3.5 as moderate loss of sensitivity, < 3.5 as severe loss of corneal sensitivity

### 4.5. Viral entry assay

HCE cells were seeded in 6-well plates at a density of approximately 5 × 10^5^ cells per well. The following day, cells were incubated with HSV-1 (McKrae strain) at a multiplicity of infection (MOI) of 10 for 40 min on ice to allow viral binding. Subsequently, cells were shifted to 37°C with 5 % CO_2_ for 15 min to facilitate viral entry. Non-internalized virions were removed by washing with a low pH buffer (50 mM sodium citrate, pH 3). Cells were then harvested and processed for western blot analysis to detect VP16 as a marker of viral entry.

An alternative assay to quantify viral entry was performed using an imaging-based approach. HCE cells were seeded in glass-bottom imaging dishes and infected with GFP-tagged HSV-1 (K26-GFP) under the same conditions, 40 min on ice followed by 15 min at 37°C with 5 % CO_2_. Cells were then fixed with 4 % paraformaldehyde (#J19943.K2; Thermo Scientific) for 15 min at room temperature. Nuclei were stained with DAPI (Thermo Scientific, #R37606), and cell membranes were labeled using Rhodamine-conjugated phalloidin (Thermo Scientific; #R415). Confocal microscopy was used to acquire multiple images, and GFP puncta representing incoming viral particles were manually quantified on a per-cell basis.

### 4.6. Plaque assay

Plaque assay was performed with tear samples from infected mice as well as with cell supernatant from in vitro experiments. Vero cell monolayers were inoculated for 2 h with virus containing sample and incubated at sample at 37°C, 5 % CO_2_. Viral suspension was aspirated and replaced with complete DMEM containing 0.5 % methyl cellulose (Fisher Scientific) for 48–72 h. Cells were then fixed with 100 % methanol and finally stained with crystal violet solution to visualize plaques.

### 4.7. Quantitative real time-polymerase chain reaction

RNA was extracted from cultured cells using TRIzol (Thermo Scientific, #15596018), following the manufacturer’s protocol, and complementary DNA was produced using High-Capacity cDNA Reverse Transcription kit (Life Technologies, # 4368814). Real-time quantitative polymerase chain reaction (qPCR) was performed using Fast SYBR Green Master Mix (Life Technologies; # 4385616) on QuantStudio 7 Flex system (Life Technologies). The following primers were used:

**Table:** 

Primer	Sequence
ICP4	F: 5′-CGGTGATGAAGGAGCTGCTGTTGC-3′
	R: 5′-CTGATCACGCGGCTGCTGTACA-3′
gB	F:5’- GCCTTTTGTGTGTGTGTGGG-3’
	R:5’- GCCTTTTGTGTGTGTGTGGG-3’
VP16	F: 5′-TCGGCGTGGAAGAAACGAGAGA-3′
	R: 5′-CGAACGCACCCAAATCGACA-3′
b-actin	F: 5′-GACGGCCAGGTCATCACTATTG-3′
	R: 5′-AGG AAGGCTGGAAAAGAGCC-3′

### 4.8. Western blot

Cellular proteins were extracted using radioimmunoprecipitation assay (RIPA; Sigma) buffer supplemented with Halt Protease and Phosphatase Inhibitor Cocktail (Thermo Scientific). Cell lysis was performed on ice with gentle agitation for 30 min, followed by centrifugation at 13,000 rpm for 20 min at 4°C to remove cellular debris. Protein concentrations in the supernatants were determined using a BCA assay. Samples were prepared by adding LDS sample loading buffer (Life Technologies) and β-mercaptoethanol (Bio-Rad, Hercules, CA), followed by denaturation at 95°C for 15 min. Proteins were resolved by SDS-PAGE on NuPAGE 4–12% Bis-Tris gels (1.5 mm, 15-well; Thermo Scientific) and transferred to PVDF membranes using the iBlot 2 transfer system (Thermo Scientific). Membranes were blocked in 5% non-fat milk in TBS-T for 1 h at room temperature and incubated with primary antibodies overnight at 4°C. After washing with TBS-T, membranes were incubated with appropriate horseradish peroxidase–conjugated secondary antibodies for 1 h at room temperature. Protein bands were visualized using SuperSignal West Femto substrate (Thermo Scientific) and imaged with an ImageQuant LAS 4000 biomolecular imager (GE Healthcare Life Sciences, Pittsburgh, PA). Actin was used as a loading control for normalization.

Antibodies used in this study are listed below:

**Table:** 

Antibody	Brand name	Catalog no.
mTOR	Proteintech	66888–1-Ig
p-mTOR	Proteintech	80596–1-AP
P70S6K	Proteintech	66638-I-Ig
pP70S6K	Proteintech	28735–AP
PI3K	Proteintech	60225-I-Ig
pPI3K	CST	4228S
AKT	Proteintech	10176–2-AP
AKT Monoclonal antibody	Proteintech	60203–2-Ig
pAKT (S473)	CST	4060S
pAKT (T308)	CST	4056S
4EBP1	Abclonal	A23500
p4EBP1	CST	9451S
Actin	Abclonal	AC026
VP16	SCBT	sc-7545
ICP4	SCBT	sc-69809
gB	SCBT	sc-56987
p-γH_2_AX	Proteintech	83307–2-RR
p53	CST	2524
Phospho-p53	CST	9284

### 4.9. Hematoxylin and eosin staining

Collected mouse tissue was first fixed with Formalin then incubated in 70 % ethanol. Paraffin embedding and sectioning using Microm HM340 was performed by the UIC histology core facilities. Subsequent H&E staining was also performed by the core facility.

### 4.10. Immunofluorescence microscopy

Cells were plated and underwent experimentation in glass bottom dishes (Cellvis; # D35–10–1.5-N). The cells were then washed with PBS and fixed using 4 % paraformaldehyde (Electron Microscopy Sciences, Hatfield, PA) for 10 min. After another wash with PBS, they were permeabilized with 0.1 % Triton X-100 (Thermo Fisher Scientific) for 15 min. The cells were washed and blocked in SuperBlock^™^ Blocking Buffer (#37515; ThermoFisher) for 1 h at room temperature. They were then incubated with primary antibodies overnight, washed using PBS, and left with a secondary antibodies conjugated with Alexa Fluor^™^ Plus 594 (#A32740 and A32742; ThermoFisher) and Alexa Fluor^™^ Plus 647 (# A32728TR and A32733TR; ThermoFisher). Nuclei were stained with NucBlue Fixed Cell ReadyProbes Reagent (#R37605; ThermoFisher). All the antibody dilutions were made in SuperBlock^™^ Blocking Buffer. The dishes were washed with PBS multiple times and then imaged using an ZEISS LSM 980 confocal microscope (Carl Zeiss). 63 × images were taken for all experiments.

### 4.11. Synergy experiments

Human corneal epithelial (HCE) cells were seeded in 96-well plates at a density of 1 × 10^4^ cells per well and infected the following day with HSV-1 K26-GFP at a multiplicity of infection (MOI) of 0.1 in serum-free medium for 2 h at 37°C. After 2 h post-infection, the viral inoculum was removed, cells were washed once with PBS, and subsequently treated with various combinations of doxorubicin and nucleoside analogs, including trifluridine, acyclovir, famciclovir, penciclovir, and valacyclovir. A checkerboard assay was employed to evaluate the combinatorial antiviral effects of doxorubicin and nucleoside analogs. HCE cells were treated with serial dilutions of each drug pair in a 96-well format. Infected, untreated cells and uninfected, drug-treated cells were included as controls. Uninfected and untreated cells served as the baseline negative control for normalization. At 24 h post-infection (hpi), fluorescence intensity corresponding to GFP expression was measured using a microplate reader (BioTek Synergy or equivalent) to assess viral replication and drug efficacy.

### 4.12. Ex vivo infection of porcine corneas

Fresh porcine eyes were obtained from a local abattoir (Peoria Packings), and corneas were excised following a previously published protocol from our laboratory (Yadavalli et al., 2021). After central epithelial debridement using a 30-gauge needle, corneas were infected with HSV-1 17-GFP. Each cornea was derived from a different animal and treated as an independent biological replicate. Beginning at 3 days post-infection (DPI), corneas were treated with doxorubicin (5 μM and 10 μM), trifluridine (TFT; 25 μM and 50 μM), a combination of doxorubicin and TFT (5 μM + 25 μM), or DMSO as a vehicle control, added to the corneal culture medium (DMEM supplemented with 1 % antibiotic–antimycotic and 10 % FBS; Sigma, U.S. origin). Unless otherwise stated, all *ex vivo* experiments were performed using corneas from at least five independent animals per condition (biological replicates). For each cornea, viral titers were quantified in duplicate technical measurements, and experiments were repeated independently twice using corneas from different animals. Ocular surface swabs were collected at 3, 5, and 7 DPI to quantify surface-associated viral titers by plaque assay. In parallel, virus released into the corneal culture medium was collected and quantified by plaque assay at the same time points. For statistical analysis, technical replicates were averaged per cornea prior to group-level comparisons.

### 4.13. Protein-ligand docking studies

Molecular docking studies for PI3Kα was performed using HerpDock (https://doi.org/10.1016/j.csbj.2024.10.013) with Doxorubicin, ACV (negative control), and positive control wherever possible. Visualization of non-bonding interaction between Protein-Ligand complexes obtained from the HerpDock was done using Discovery Studio (BIOVIA, D. S. Discovery Studio Visualizer, v19. 1.0. 1828 (2019). San Diego: Dassault Systèmes).

### 4.14. MTT assay

MTT viability assays were performed to assess the cytotoxicity of doxorubicin across various primary and immortalized cell lines at different concentrations after 24 and 48 h of incubation. Primary cell lines included mouse primary adult dermal fibroblasts (MAFs) and pig corneal fibroblasts (PCFs), while immortalized cell lines included HeLa, HCE, mouse embryonic fibroblasts (MEFs), and Vero cells. Briefly, cells were seeded in 96-well plates at a density of 1 × 10^4^ cells per well and incubated overnight. The following day, doxorubicin was added to the wells in two-fold serial dilutions in complete media and incubated for 24 or 48 h. After the incubation period, MTT reagent (0.5 mg/mL in complete media) was added to each well and incubated for 3 h to allow formazan crystal formation. Following incubation, acidified isopropanol (1 % glacial acetic acid v/v) was added to solubilize the crystals. The resulting solution was transferred to a fresh 96-well plate, and absorbance was measured at 570 nm using a microplate reader. Cell viability was expressed as a percentage relative to untreated control cells.

### 4.15. Corneal fluorescein staining

Corneal epithelial integrity was assessed by fluorescein staining at day 8 post ocular HSV-1 infection. Mice were divided into six groups: non-infected mock treated, non-infected treated with trifluridine (TFT), non-infected treated with doxorubicin, HSV-1 infected mock treated, HSV-1 infected treated with TFT, and HSV-1 infected treated with doxorubicin. Prior to staining, mice received a systemic anesthetic, an intraperitoneal injection of ketamine (100 mg/kg) and xylazine (5 mg/kg). Sodium fluorescein (0.1 % w/v in sterile PBS) was applied to the corneal surface (5 μL per eye), allowed to distribute for 10–20 s, and gently rinsed with sterile PBS to remove excess dye. Corneas were examined under stereomicroscope, and images were captured under identical exposure settings for all groups. All procedures were performed at the same time point to minimize variability across experimental groups.

### 4.16. Masson’s trichrome staining of heart tissue

Cardiac fibrosis and tissue architecture were evaluated using Masson’s trichrome staining. Hearts were harvested, rinsed in cold phosphate-buffered saline, and fixed in 10 % neutral buffered formalin for 24–48 h at room temperature. Fixed tissues were processed, paraffin embedded and sectioned at 5 μm thickness. Sections were deparaffinized, rehydrated through graded ethanol, and stained using a standard Masson’s trichrome kit available from Abcam (Catalogue No.: ab150686) by following manufacturer’s instructions.

### 4.17. Complete blood count analysis

Blood was collected from mice at 8 days post infection for complete blood count analysis. Whole blood was obtained by cardiac puncture into EDTA-coated tubes to prevent coagulation. Samples were gently inverted to ensure proper mixing and analyzed within 2 h of collection. Complete blood counts, including total white blood cell count, red blood cell count, hemoglobin concentration, hematocrit, platelet count, and differential leukocyte percentages, were measured using an automated hematology analyzer calibrated for murine blood by BRL at UIC.

### 4.18 TUNEL staining

Apoptotic cell death was assessed using terminal deoxynucleotidyl transferase dUTP nick end labeling (TUNEL) staining. Paraffin and OCT embedded tissue (Eye, TG, Brain, Optic Nerve and Heart) sections (5 μm) were deparaffinized, rehydrated through graded ethanol, and subjected to antigen retrieval or proteinase K treatment according to the manufacturer’s instructions. TUNEL staining was performed using CoraLite^®^594 TUNEL Assay Apoptosis Detection Kit from Proteintech (Cat No. PF00009) according to the manufacturer’s instructions.

## Supplementary Material

1

## Data Availability

All data are available in the main text or the supplementary materials

## Appendix A. Supporting information

Supplementary data associated with this article can be found in the online version at doi:10.1016/j.drup.2026.101362.
