# Supplementary material for: Host-directed novel mechanistic insights of doxorubicin reveal its efficacy against drug-resistant HSV-1 underscoring risks with oncolytic virotherapy
Source: Drug Resist Updat. Author manuscript; Available in PMC 2026 Jul 14. (PMC13367419; doi:10.1016/j.drup.2026.101362)
Supplement: 1 [file NIHMS2190249-supplement-1.docx]

**Supplementary Figures:**

**
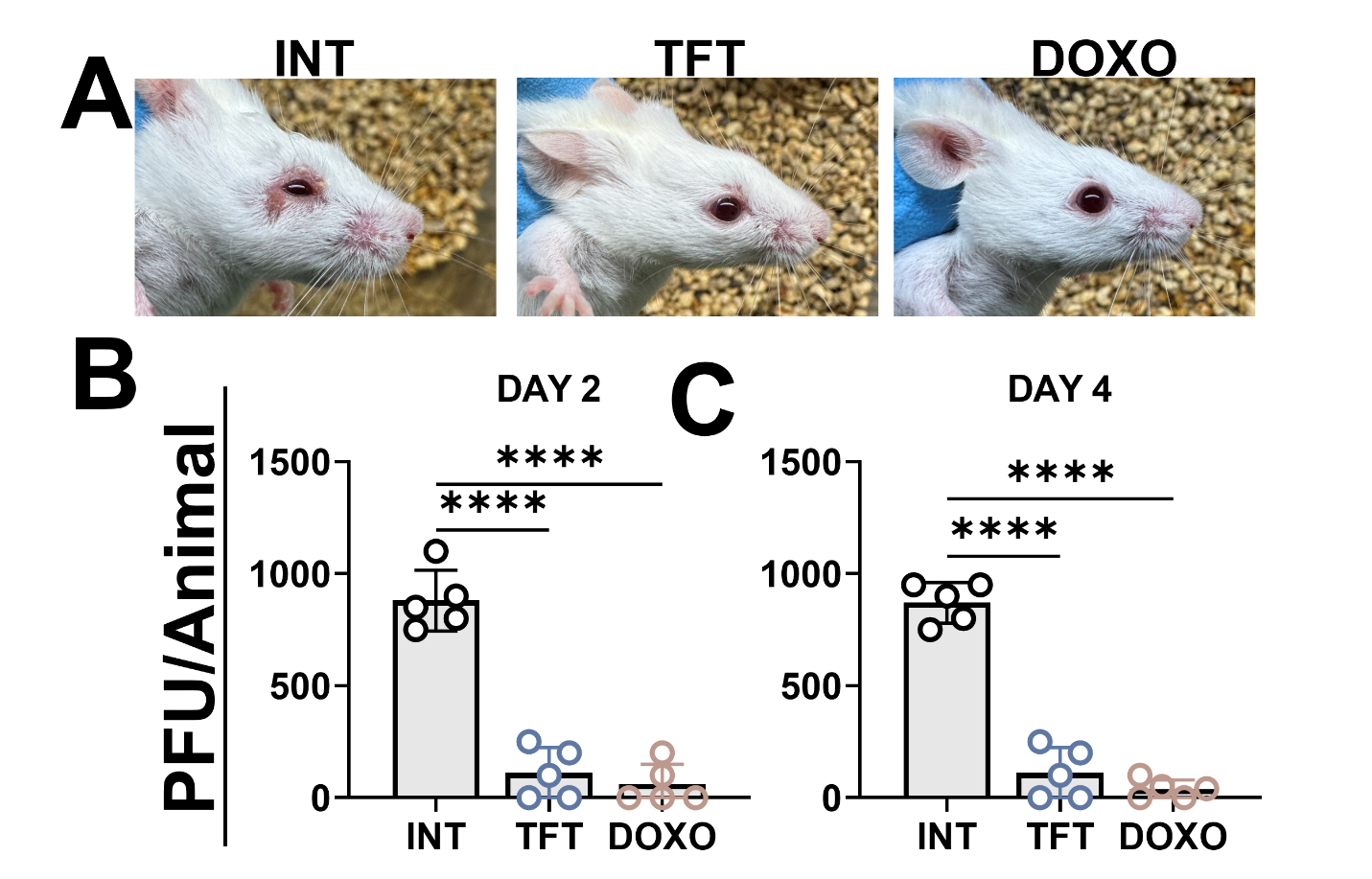
**

**Fig. S1. Topical treatment with doxorubicin reduces ocular infection of oncolytic HSV-1 in mice.**
(A) Representative images of mice from the infected non-treated (I-NT), trifluridine (TFT)-treated, and doxorubicin-treated groups, collected at 8 days post-infection (DPI).
(B) Plaque assays of eye washes collected at 2 DPI showing reduced viral titers following topical treatment with TFT and doxorubicin.
(C) Plaque assays of eye washes collected at 4 DPI demonstrating continued reduction in viral titers in the TFT- and doxorubicin-treated groups compared to the non-treated control. Statistical analysis was conducted using one-way ANOVA.


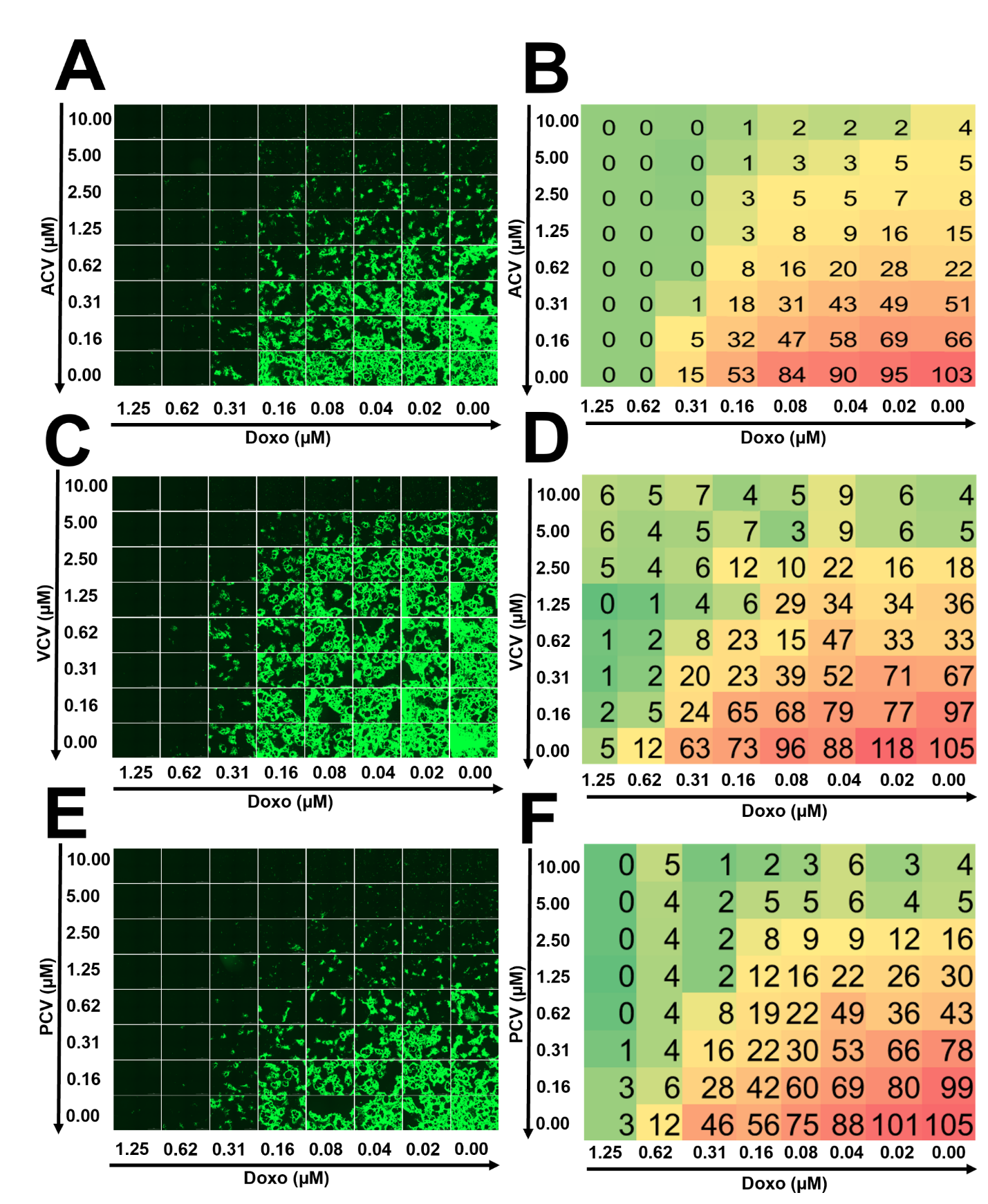


**Fig. S2. Checkerboard assays reveal synergistic enhancement of nucleoside analog activity by doxorubicin in HSV-1–infected HCE cells.**

(A) HCE cells infected with HSV-1 K26-GFP at a multiplicity of infection (MOI) of 0.1 were treated with acyclovir (ACV) and doxorubicin at varying concentrations, individually and in combination, using an 8 × 8 checkerboard format to assess drug synergy.

(B) GFP fluorescence intensity from (A) was quantified using a plate reader at 24 hours post-infection to evaluate antiviral efficacy.

(C) HCE cells infected with HSV-1 K26-GFP (MOI 0.1) were treated with valacyclovir (VCV) and doxorubicin in a similar 8 × 8 checkerboard format.

(D) Quantification of GFP fluorescence intensity from (C) was performed using a plate reader at 24 hours post-infection.

(E) HCE cells infected with HSV-1 K26-GFP (MOI 0.1) were treated with penciclovir (PCV) and doxorubicin across a range of concentrations in an 8 × 8 checkerboard layout.

(F) GFP fluorescence intensity from (E) was measured using a plate reader at 24 hours post-infection to assess antiviral synergy.


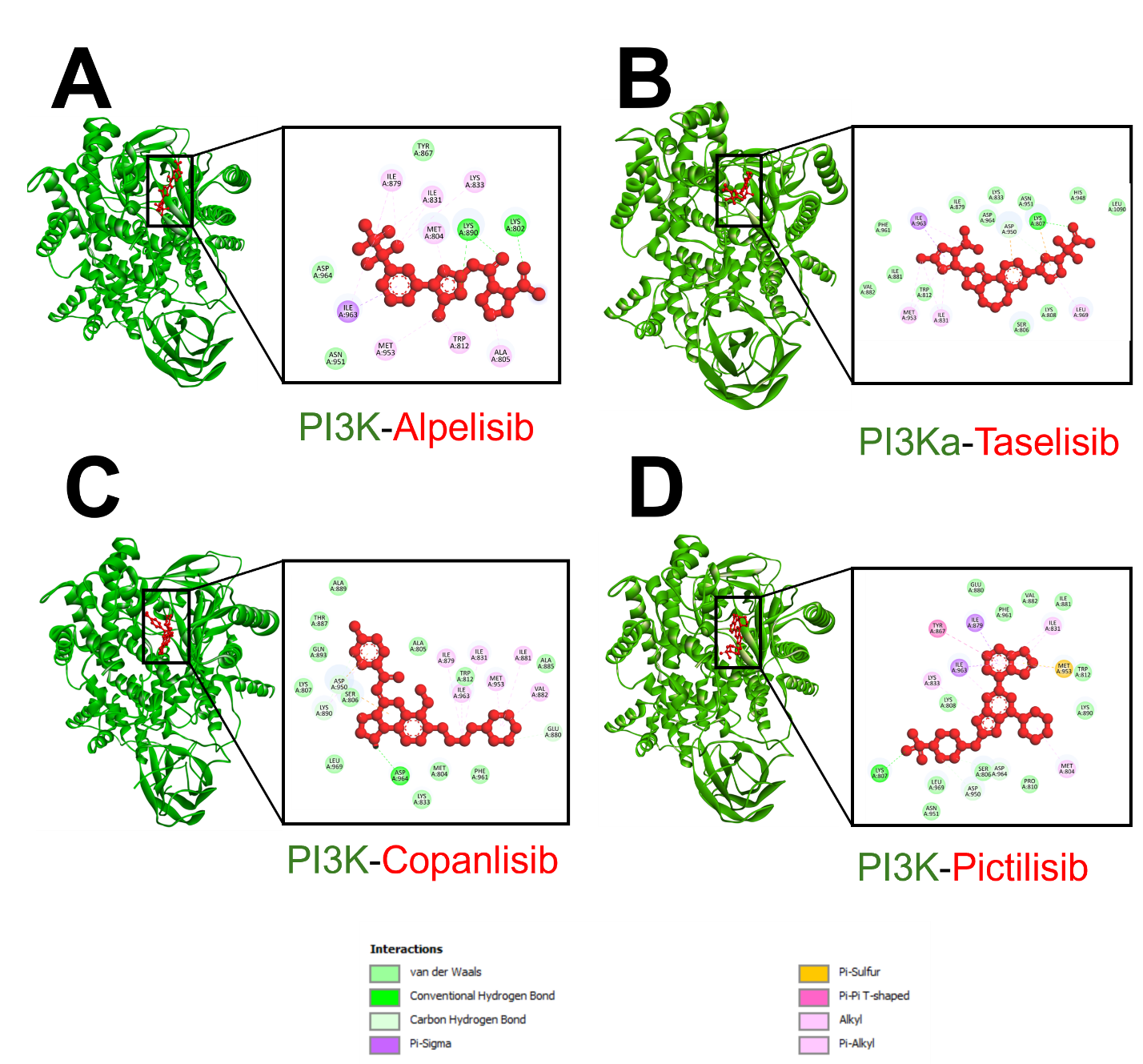


**Fig. S3. In silico docking analysis showing interactions between PI3K and different inhibitors.**
(A) In silico molecular docking analysis illustrating the interaction between Alpelisib (ball-and-stick representation) and PI3K (cartoon representation), performed using Discovery Studio.
(B) Docking analysis showing the interaction between Taselisib (ball-and-stick) and PI3K (cartoon), performed using Discovery Studio.
(C) Molecular docking analysis depicting the interaction between Copanlisib (ball-and-stick) and PI3K (cartoon), generated in Discovery Studio.
(D) Docking visualization of Pictilisib (ball-and-stick) bound to PI3K (cartoon), performed using Discovery Studio.


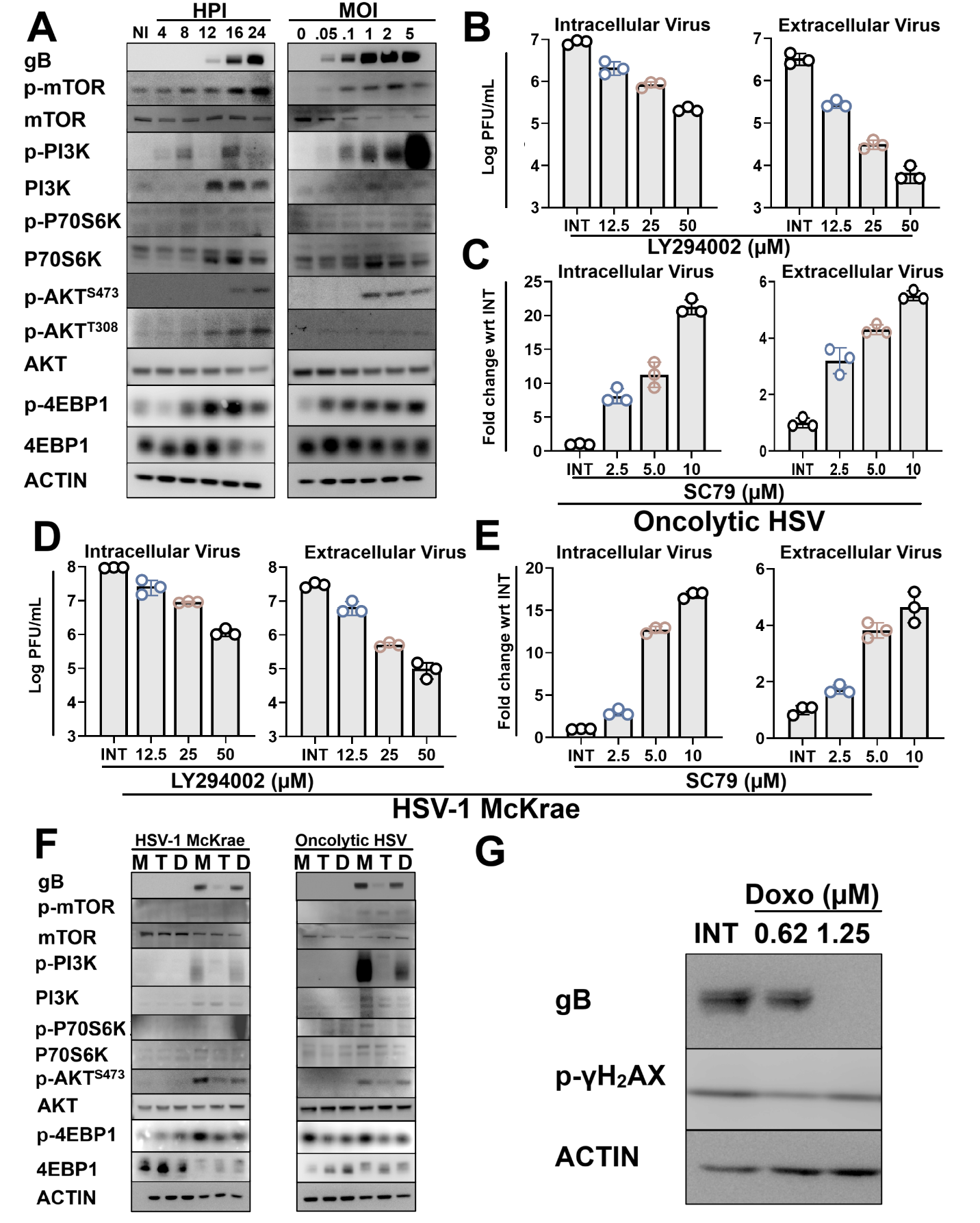


**Fig. S4. Productive replication of oncolytic HSV requires activation of the PI3K–AKT signaling axis.**(A) Immunoblot analysis demonstrating time- and dose-dependent activation of the PI3K–AKT pathway during oncolytic HSV infection.
(B, C) Pharmacological inhibition of PI3K using LY294002 suppresses oncolytic HSV infection, whereas pharmacological activation of AKT using SC79 enhances viral replication, indicating a critical role for the PI3K-AKT axis in supporting oncolytic HSV replication.
(D, E) Pharmacological inhibition of PI3K using LY294002 similarly restricts infection with wild-type HSV-1 (McKrae strain), while AKT activation using SC79 promotes viral replication, supporting a conserved requirement for PI3K-AKT signaling during HSV infection.
(F) Doxorubicin-mediated inhibition of the PI3K-AKT pathway in the doxorubicin-resistant H69AR cell line results in reduced viral load, demonstrating that the antiviral activity of doxorubicin is independent of topoisomerase II inhibition and instead reflects host-directed suppression of PI3K-AKT signaling.
(G) Immunoblot analysis showing reduced phosphorylation of γH_2_AX, a marker of DNA damage and topoisomerase II inhibition. The absence of γH_2_AX induction supports the conclusion that doxorubicin’s antiviral activity under these conditions is mediated primarily through inhibition of the PI3K-AKT pathway rather than genotoxic stress.
